# Mutation in Polycomb repressive complex 2 gene *OsFIE2* promotes asexual embryo formation in rice

**DOI:** 10.1038/s41477-023-01536-4

**Published:** 2023-10-09

**Authors:** Xiaoba Wu, Liqiong Xie, Xizhe Sun, Ningning Wang, E. Jean Finnegan, Chris Helliwell, Jialing Yao, Hongyu Zhang, Xianjun Wu, Phil Hands, Falong Lu, Lisong Ma, Bing Zhou, Abed Chaudhury, Xiaofeng Cao, Ming Luo

**Affiliations:** 1 CSIRO Agriculture and Food, Canberra, Australian Capital Territory Australia; 2 Xinjiang Key Laboratory of Biological Resources and Genetic Engineering, School of Life Science and Technology, Xinjiang University, Urumqi, P. R. China; 3 The State Key Laboratory of North China Crop Improvement and Regulation, College of Horticulture, Hebei Agricultural University, Baoding, P. R. China; 4 Division of Plant Science, Research School of Biology, the Australian National University, Canberra, Australian Capital Territory Australia; 5 Faculty of Agronomy, Jilin Agricultural University, Changchun, P. R. China; 6 College of Life Science and Technology, Huazhong Agricultural University, Wuhan, P. R. China; 7 State Key Laboratory of Crop Gene Exploration and Utilization in Southwest China, Rice Research Institute, Sichuan Agricultural University, Chengdu, P. R. China; 8 State Key Laboratory of Molecular Developmental Biology, Institute of Genetics and Developmental Biology, Chinese Academy of Sciences, Beijing, P. R. China; 9 University of Chinese Academy of Sciences, Beijing, P. R. China; 10 Institute of Zoology, Chinese Academy of Sciences, Beijing, P. R. China; 11 Krishan Foundation Pty Ltd, Canberra, Australian Capital Territory Australia; 12 State Key Laboratory of Plant Genomics and National Center for Plant Gene Research, Institute of Genetics and Developmental Biology, Chinese Academy of Sciences, Beijing, P. R. China

**Keywords:** Imprinting, Plant embryogenesis

## Abstract

Prevention of autonomous division of the egg apparatus and central cell in a female gametophyte before fertilization ensures successful reproduction in flowering plants. Here we show that rice ovules of Polycomb repressive complex 2 (PRC2) *Osfie1* and *Osfie2* double mutants exhibit asexual embryo and autonomous endosperm formation at a high frequency, while ovules of single *Osfie2* mutants display asexual pre-embryo-like structures at a lower frequency without fertilization. Earlier onset, higher penetrance and better development of asexual embryos in the double mutants compared with those in *Osfie2* suggest that the autonomous endosperm facilitated asexual embryo development. Transcriptomic analysis showed that male genome-expressed *OsBBM1* and *OsWOX8/9* were activated in the asexual embryos. Similarly, the maternal alleles of the paternally expressed imprinted genes were activated in the autonomous endosperm, suggesting that the egg apparatus and central cell convergently adopt PRC2 to maintain the non-dividing state before fertilization, possibly through silencing of the maternal alleles of male genome-expressed genes.

## Abstract (web summary)

Wu et al. show that mutations of the Polycomb repressive complex 2 gene, *OsFIE2*, result in asexual embryo formation in rice, suggesting a critical role of the epigenetic regulation in maintaining the quiescent state of the egg apparatus.

## Main

In flowering plants, seed propagation requires double fertilization, in which the haploid egg cell and the homodiploid central cell in a female gametophyte (embryo sac) are fertilized with two genetically identical sperms to form a diploid embryo and triploid endosperm^1,2^. The fertilized egg or zygote starts embryogenesis following a serial division stage to form a basic body plan for a stereotyped seedling. Genes with de novo expression in the fertilized egg (zygote) are important for embryogenesis^2–4^. Among these genes with known embryogenic functions in rice are transcription factors, such as *OsBBM1*, *OsBBM2*, *OsWOX8/9* and *OsWOX2* (refs. ^4–7^). *OsBBM1*, *OsBBM2* and *OsWOX8/9* are specifically expressed from the paternal alleles after fertilization and ectopic expression of *OsBBM1* in the egg can trigger embryogenesis^3,4^, suggesting that the imprinted state of the maternal allele is important for egg quiescence. Little is known about the mechanism that maintains this quiescent state before fertilization.

The fertilized central cell nucleus starts to divide without cytokinesis to form a multinucleate cell called syncytial endosperm, followed by cellularization. A body of knowledge that explains how the central cell is prevented from autonomous division has been built up in *Arabidopsis* and rice^8–15^. Mutations in members of the Polycomb repressive complex 2 (PRC2) genes cause autonomous endosperm development potentially by activating the auxin biosynthesis pathway in the central cell in the absence of fertilization^16,17^. PRC2, an important epigenetic regulator, catalyses histone H3 lysine27 trimethylation (H3K27me^3^)^17,18^, which regulates imprinting in endosperm by preferentially targeting maternal alleles of paternally expressed imprinted genes (PEGs)^18–24^.

Despite progress in understanding the role of PRC2 in regulating endosperm development in rice, studies on how the two PRC2 FERTILIZATION INDEPENDENT ENDOSPERM (FIE) homologues suppress central cell initiation report inconsistent results^14,25–27^. By analysing multiple mutants of the closely linked *Osfie1* and *Osfie2* mutations induced via CRISPR/Cas9, we uncovered a novel function of PRC2 in suppressing cell division of the egg apparatus in the absence of fertilization, and provided further insight on the role of the *OsFIE* genes in reproductive development. Transcriptomic analysis revealed the activation of pluripotency factor genes *OsBBM1* and *OsWOX8/9* in asexual embryos and PEGs in autonomous endosperm. This study provides evidence of the pivotal roles of PRC2 in maintaining the repressive state of the egg (or egg apparatus) and central cell from autonomous division to ensure reproductive integrity in rice.

## Results

### CRISPR/Cas9-induced mutants at *OsFIE1* and *OsFIE2*

**Fig. 1:**
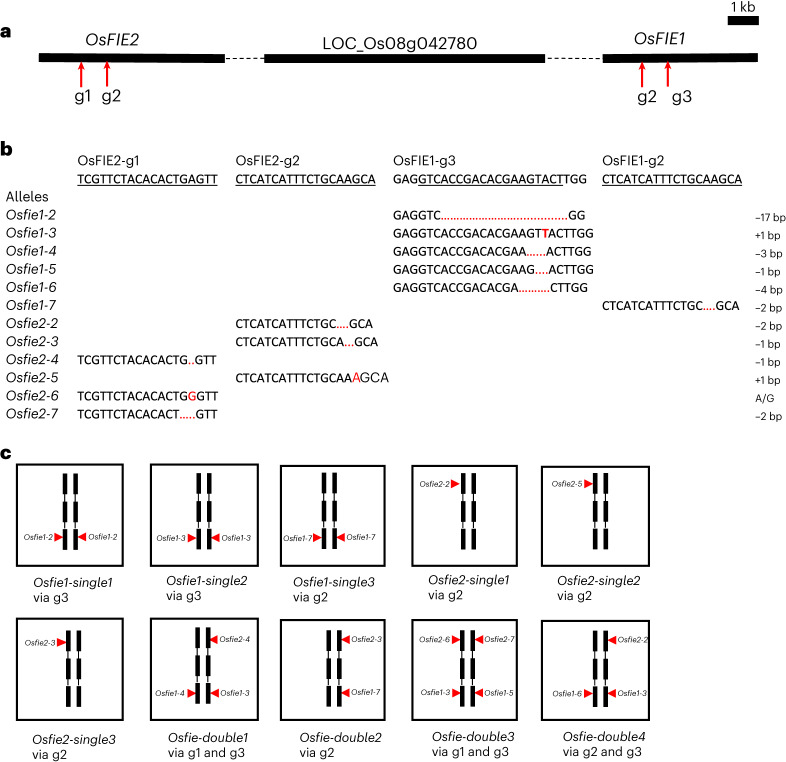
Mutant alleles and genotypes at *OsFIE* loci induced by the CRISPR/Cas9 system. **a**, Positions at the exons of two *OsFIE* loci targeted by three gRNAs (g1, g2 and g3), two gRNAs targeting *OsFIE1* and *OsFIE2*, respectively (g1 and g3) and one targeting an identical region between two genes (g2); *OsFIE1* is ~9 kb away from *OsFIE2*. **b**, Mutant alleles induced by CRISPR/Cas9 at the two gene loci; black underline indicating gRNA target; red dotted line indicating the deletion and red letter indicating addition or substitution. **c**, Genotypes and associated gRNA targets (g1, g2 and g3) of three *Osfie1*, three *Osfie2* and four double mutants used for analysis (Table 1).

**Fig. 2:**
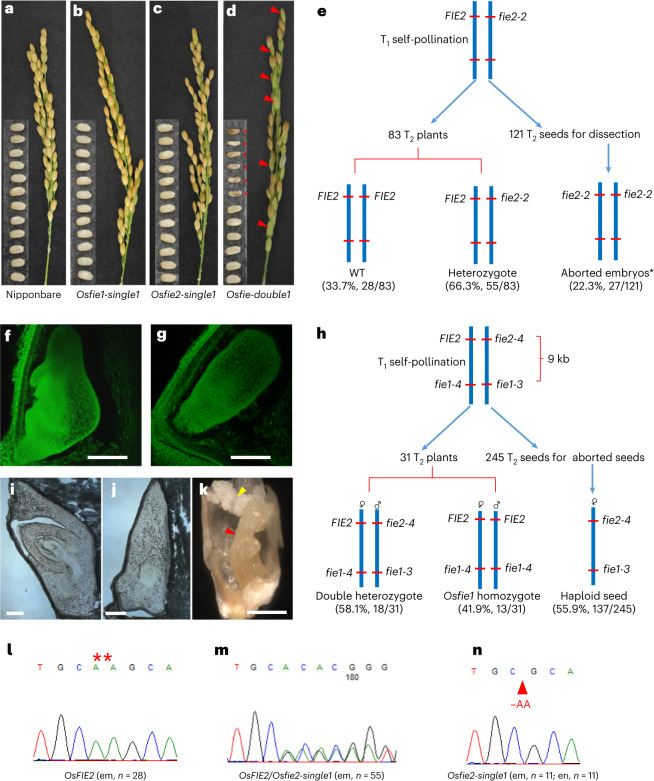
Seed phenotypes and genetic segregation of the self-pollinated *Osfie* mutants. **a**–**d**, Panicles with de-husked seeds: Nipponbare (**a**), *Osfie1-single1* homozygote (**b**), *Osfie2-single1* heterozygote (**c**) and *Osfie-double1* heterozygote (**d**). Red asterisks show the aborted seeds; red arrowheads show the spiklets containing aborted seeds. **e**, T_2_ plants (83) from an *Osfie2-single1* T_1_ heterozygote segregated into two genotypes: WT and heterozygote at a ~1:2 (28:55) ratio. Hand dissection of 121 T_2_ seeds shows that ~25% (27/121) had aborted embryos, which were considered to be *Osfie2* homozygotes (Table 1). **f**, Normal 5-day-old embryo from an *Osfie2-single1* heterozygote under confocal microscopy (*n* = 3). **g**, Abnormal 5-day-old embryo from an *Osfie2-single1* heterozygote under confocal microscopy (*n* = 3). **h**, T_2_ plants (31) from an *Osfie-double1* T_1_ heterozygote segregated into two genotypes: the double heterozygotes similar to the parent showing aborted seeds and *Osfie1* homozygous segregants showing no aborted seeds with a ~1:1 (18:13) ratio. Detailed phenotyping of the aborted seeds by dissection shows ~50% (137/245) collapsed seeds containing aborted embryos (**k**), which were shown to be haploid (Fig. 5b–f). **i**, Well-differentiated embryos from *Osfie2-single1* heterozygote by cryo-section at maturation (*n* = 3). **j**, Abnormal embryos from *Osfie2-single1* heterozygote by cryo-section at maturation (*n* = 3). **k**, Aborted seeds from *Osfie-double1* at maturation showing deformed embryo (red arrowhead) and endosperm (yellow arrowhead) (*n* = 137). **l**, Sanger sequencing of segregants (*n* = 28) of *Osfie2-single1* heterozygote showing the WT allele at *OsFIE2*; red asterisks show 2 bp ‘AA’, which are deleted at the *Osfie2-2* locus. **m**, Sanger sequencing of segregants (*n* = 55) of *Osfie2-single1* heterozygote showing the heterozygote allele at *OsFIE2*. **n**, Sanger sequencing of embryos and endosperm (*n* = 11) of non-germinated seeds from *Osfie2-single1* heterozygote showing the mutant allele with two base-pair deletion (−AA, red arrowhead) at the *Osfie2-2* locus. Scale bars, 50 μm.

**Extended Data Fig. 1:**
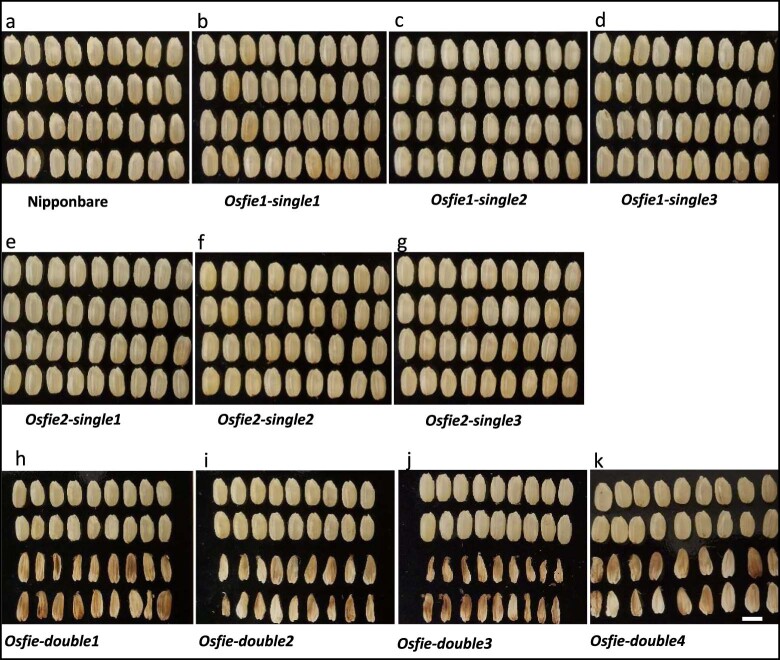
De-husked seeds of WT and self-pollinated *Osfie* mutants. **a**, Seeds from Nipponbare (WT). **b–d**, Seeds from *Osfie1-single1*, *2*, and *3* homozygotes. **e–g**, Seeds from *Osfie2-single1*, *2*, and *3* heterozygotes. **i–k**, Seeds from *Osfie-double1*, *2*, *3* and *4* heterozygotes (Bar = 0.5mm for all figures).

Among ~150 T_0_ plants transformed with the CRISPR/Cas9 vector targeting the two *FIE* genes (Fig. 1a), we focused on plants with normal pollen dehiscence, as heterozygous *fie* mutants and other Polycomb mutants are not expected to give pollen sterility in heterozygous condition^7–14^. Four plants that showed distinct ~50% shrivelled seed formation without other morphological phenotypes were isolated as *Osfie* double or *Osfie2* mutant candidates on the basis of phenotypic similarity to previously reported Polycomb mutants. Among other normal-looking plants, with random Sanger sequencing, we identified three putative homozygous *Osfie1* (Fig. 1b,c and Table 1; designated as *Osfie1-single1*, *2* and *3*) and three putative heterozygous *Osfie2* mutants at the expected positions (Fig. 1b,c and Table 1; designated as *Osfie2-single1*, *2* and *3*). The transgene-free homozygous *Osfie1* plants isolated at T_1_ displayed no apparent phenotype as expected^25^ (Fig. 2a,b and Extended Data Fig. 1a–d). The transgene-free *Osfie2* heterozygotes at T_1_ also had normal seed formation and set comparable to wild-type Nipponbare (Fig. 2c and Extended Data Fig. 1e–g), in contrast to the previous finding where an *Osfie2* mutation caused seeds to shrivel^14^. By analysing T_2_ progeny derived from the transgene-free T_1_ heterozygotes of *Osfie2-single1*, we could not find homozygous mutants but found wild type (WT) and heterozygotes segregating at ~1:2 ratio (28:55) in the progeny (Table 1 and Fig. 2e,l,m), suggesting that the *Osfie2* mutations probably caused homozygous lethality of embryos without causing seed shrivelling. We further analysed germinating seeds harvested from the transgene-free heterozygote of *Osfie2-single1* to verify the embryo lethal phenotype. The *OsFIE2* PCR fragments from the embryo and endosperm of non-germinated seeds were sequenced and showed that many of these seeds were homozygous for the *Osfie2-2* mutation (Fig. 2n). The self-pollinated seeds of *Osfie2-single1* containing arrested embryos were confirmed by confocal microscopy of developing seeds and cryo-section of mature seeds (Fig. 2f,g,i,j). We further hand sectioned the mature seeds from the heterozygote and found that ~25% of seeds (27/121) had abnormally differentiated embryos, while the other ~75% of the embryos appeared normal (Table 1 and Fig. 2e). Similarly, another two *Osfie2* single mutants displayed similar genetic segregation and embryo abortion phenotype at T_2_, lacking homozygous mutant segregants (Table 1). This result suggests that *OsFIE2* functions alone in embryo development but redundantly with *OsFIE1* in endosperm development, suggesting that there is partial subfunctionalization between *OsFIE1* and *OsFIE2*, leading to differential controls on embryo development (only by *OsFIE2*) and endosperm development (by both *FIE* genes).

**Table 1:** Summary of the genotypes and phenotypes of the *Osfie* single and double mutants

Mutant names	Mutations	Genotypes in T_2_ or T_4_^a^	Seed abortion	Autonomous phenotype^d^	Asexual embryos^e^
*Osfie1-single1*	*Osfie1-2* (−17 bp)	*Osfie1-2/Osfie1-2*		0 (*n* = 15)	
*Osfie1-single2*	*Osfie1-3* (+T)	*Osfie1-3/Osfie1-3*		0 (*n* = 26)	
*Osfie1-single3*	*Osfie1-7* (−AA)	*Osfie1-7/Osfie1-7*		0 (*n* = 39)	
*Osfie2-single1*	*Osfie2-2* (−AA)	*OsFIE2/Osfie2-2* (*n* = 55); WT (*n* = 28)	27 (22.3%, *n* = 121)^b^		9 (21.4%, *n* = 42) at T_2_ 22 (22.2%, *n* = 99) at T_3_
*Osfie2-single2*	*Osfie2-5*(+A)	*OsFIE2/Osfie2-5* (*n* = 4); WT (*n* = 2)	72 (33.6%, *n* = 214)^b^		6 (18.2%, *n* = 33) at T_2_ 5 (22.7%, *n* = 22) at T_3_
*Osfie2-single3*	*Osfie2-3*(−A)	*OsFIE2/Osfie2-3* (*n* = 34, 63.0%); WT (*n* = 20, 37%)	37 (13.4%, *n* = 276)^b^		10 (17.86%, *n* = 56) at T_2_ 12 (19.0%, *n* = 63) at T_3_
*Osfie-double1*	*Osfie1-3* (+T); *Osfie1-4* (−GTA); *Osfie2-4* (−A)	*Osfie1-4/Osfie1-3 OsFIE2/Osfie2-4* (*n* = 18); *Osfie1-4/Osfie1-4* (*n* = 13)	137 (55.9%, *n* = 245)^c^	103 (46.6%, *n* = 221) at T_2_	33 (37.9%, *n* = 87) at T_2_
*Osfie-double2*	Osfie1-7 (−AA); *Osfie2-3* (−A)	*OsFIE1/Osfie1-7 OsFIE2/Osfie2-3* (*n* = 13); WT (*n* = 16)	126(62.3%, *n* = 202)^c^	53 (53.5%, *n* = 99) at T_4_	39 (50.6%, *n* = 77) at T_4_
*Osfie-double3*^f^	*Osfie1-3* (+T); *Osfie1-5* (−T); *Osfie2-6* (A/G); *Osfie2-7* (−GA)	*Osfie1-3/Osfie1-5 Osfie2-6/Osfie2-7* (*n* = 3); *Osfie1-3/Osfie1-3 Osfie2-6/Osfie2-6* (*n* = 3)	23 (56.1%, *n* = 41)^c^	20 (47.6%, *n* = 42) at F_1_ (WT × T_3_)	13 (31.0%, *n* = 42) at F_1_ (WT × T_3_)
*Osfie-double4*^f^	*Osfie1-3* (+*T); Osfie1-6* (−*AGTA); Osfie2-2* (−*AA)*	*Osfie1-6/Osfie1-3 OsFIE2/Osfie2-2* (*n* = *10)*; *Osfie1-6/Osfie1-6* (*n* = *6)*	27 (64.3%, *n* = 42)^c^	40 (51.3%, *n* = 78) at F_1_ (WT × T_3_)	26 (33.3%, *n* = 78) at F_1_ (WT × T_3_)

^a^Genotypes of transgene-free T_2_ (T_4_ is for *Osfie-double2*) plants by Sanger sequencing.

^b^Abortion ratio based on self-pollinated seeds harvested from transgene-free T_1_ for *Osfie2-single2* and *Osfie2-single3*, and T_2_ for *Osfie2-single1*. Seeds were cut in transection through embryo and observed under a stereomicroscope to visualize the aborted embryo (as shown in Fig. 2).

^c^Aborted seeds were scored as shrivelled seeds (as shown in Fig. 3) for double mutants at T_2_ (T_4_ for *Osfie-double2*).

^d^For *Osfie* double mutants, autonomous phenotypes were scored with ovules containing embryo-like structures and/or endosperm 0 d after emasculation (Supplementary Table 1).

^e^For *Osfie2-single* mutants, asexual embryos were scored with ovules containing pre-embryo-like structures >7 d after emasculation (Supplementary Table 1). For *Osfie* double mutants, asexual embryos were scored with ovules containing embryo-like structure >5 d after emasculation (Supplementary Table 1).

^f^F_1_ progeny of WT pollinated with *Osfie-double3* or *Osfie-double4* were used for scoring the autonomous phenotype and asexual embryos (Supplementary Table 1).

**Extended Data Fig. 2:**
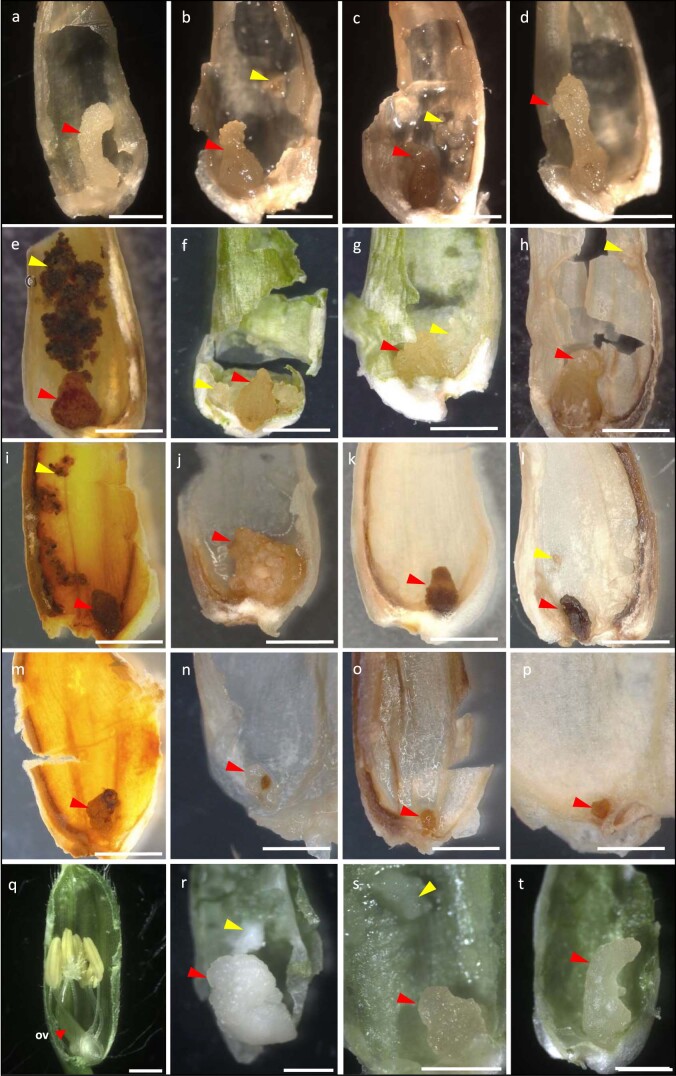
Aborted embryos and endosperm in *Osfie* double mutants under self-pollination or emasculation. **a–d**, Self-pollinated *Osfie-double1* at maturity; n = 137 aborted seeds dissected. **e–h**, Self-pollinated *Osfie-double2* at maturity; n = 126 aborted seeds dissected. **i–l**, Self-pollinated *Osfie-double3* at maturity; n = 23 aborted seeds dissected. **m**–**p**, Self-pollinated *Osfie-double4* at maturity; n = 27 aborted seeds dissected. **q**, Slightly elongated ovules with intact anthers from *Osfie-double1* on the day for emasculation (n = 4). **r**–**t**, Asexual embryos and autonomous endosperm in *Osfie-double1* at 15 DPE. Noting that endosperm is visually absent in t; n = 17 aborted seeds dissected. In all figures except **q**, the red arrowheads indicate embryos and yellow arrowheads indicate endosperm; In **q**, red arrowhead indicates ovary (ov); Scale bar = 0.5mm.

For the four T_0_ plants showing ~50% seed shrivelling, we found simultaneous mutations at both *OsFIE1* and *OsFIE2* loci (Figs. 1c and 2d, and Table 1; designated as *Osfie-double1*, *2*, *3* and *4*), with three having heteroallelic mutations and one being heterozygous at *OsFIE1*. All the *Osfie1* mutations were predicted to cause loss of function. At *OsFIE2*, three plants were heterozygous and all the mutations were predicted to cause loss of function, while the fourth plant (termed *Osfie-double3*) had heteroallelic mutations (Fig. 1c), with *Osfie2-7* being predicted to cause loss of function and *Osfie2-6* predicted to be a weak allele that retains function, as it resulted in a serine to glycine substitution in a non-conserved region. We speculated that if *Osfie2-6* caused loss of function, the heteroallelic *Osfie2-6/Osfie2-7* genotype would have caused embryo lethality and this plant would not have been isolated. We selected transgene-free mutants at T_1_, which all showed shrivelled seed phenotypes (Extended Data Fig. 1h–k). From the mutant *Osfie-double1* (Fig. 1c), we could identify 13 plants homozygous for *Osfie1-4* showing no shrivelled seeds, and 18 plants heterozygous for *Osfie2-4* and heteroallelic for *Osfie1* displaying similar shrivelled seeds as the T_1_ parent, without double homozygous mutants being identified, suggesting that the linked loss-of-function mutations at both *OsFIE* loci could not be transmitted to T_2_ by the mutant female gametophyte (Fig. 2h). The double mutations could be transmitted by pollen and the mutant ovules carrying double mutations gave aborted seed upon fertilization (see below) (Table 1 and Fig. 2h). Using a progeny of the double mutant at T_2_, we confirmed the 1:1 segregation of the two genotypes at T_3_, with no other genotypes being identified. Similar segregation and seed abortion were seen in the other three double mutants at T_4_ for *Osfie-double2* and T_2_ for *Osfie-double3* and *4* (Table 1 and Fig. 1c). By dissecting the aborted seeds, we observed abnormally differentiated embryos of various morphologies, many with aborted endosperm (Fig. 2k and Extended Data Fig. 2a–p). The closely linked *fie* mutations caused seed abortion in a maternal gametophytic manner, a similar phenotype observed in the *Arabidopsis* *FIE* and other *fis*-class mutants^9^.

### Asexual embryos and autonomous endosperm in double mutants

**Fig. 3:**
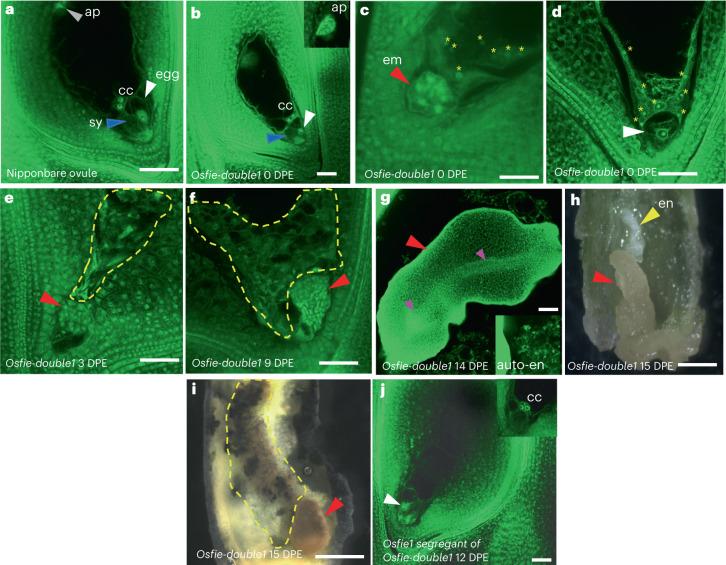
Asexual embryo and autonomous endosperm development in *Osfie-double1*. **a**, Ovules of Nipponbare, with an egg (white arrowhead), two synergids (sy, one in focus by blue arrowhead), two central nuclei (cc) and antipodal cells (ap, grey arrowhead) at 0 DPE (*n* = 35). **b**, WT-looking ovules of *Osfie-double1*, with an egg (white arrowhead), two synergids (one in focus by blue arrowhead), two central nuclei and antipodal cells (insert) at 0 DPE (*n* = 31). **c**, Asexual globular embryos (em, red arrowhead) and syncytial endosperm (yellow asterisks) in *Osfie-double1* at 0 DPE (*n* = 6). **d**, Ovules with autonomous endosperm (yellow asterisks) in *Osfie-double1* at 0 DPE, with an egg cell (white arrowhead) (*n* = 26). **e**, Asexual globular embryos (red arrowhead) and cellularized endosperm (circled by yellow dashed line) in *Osfie-double1* at 3 DPE (*n* = 5). **f**, Asexual globular embryos (red arrowhead) and cellularized endosperm (circled by yellow dashed line) in *Osfie-double1* at 9 DPE (*n* = 17). **g**, Asexual embryos (red arrowhead) and autonomous endosperm (insert) in *Osfie-double1* at 14 DPE, showing vascular structure (pink arrowheads) (*n* = 9). **h**, Asexual embryo (red arrowhead) and autonomous endosperm (en, yellow arrowhead) in a dissected seed of *Osfie-double1* at 15 DPE (*n* = 17). **i**, Asexual embryo (red arrowhead) and autonomous endosperm (circled by yellow dashed line) in a dissected seed of *Osfie-double1* at 15 DPE, showing starch granules (dark) being stained with iodine potassium-iodide solution (*n* = 3). **j**, Embryo sacs with an egg (white arrowhead) and central cell (insert) of an *Osfie1* segregant of *Osfie-double1* at 12 DPE, showing no autonomous development (*n* = 22). Scale bars, 50 μm.

**Extended Data Fig. 3:**
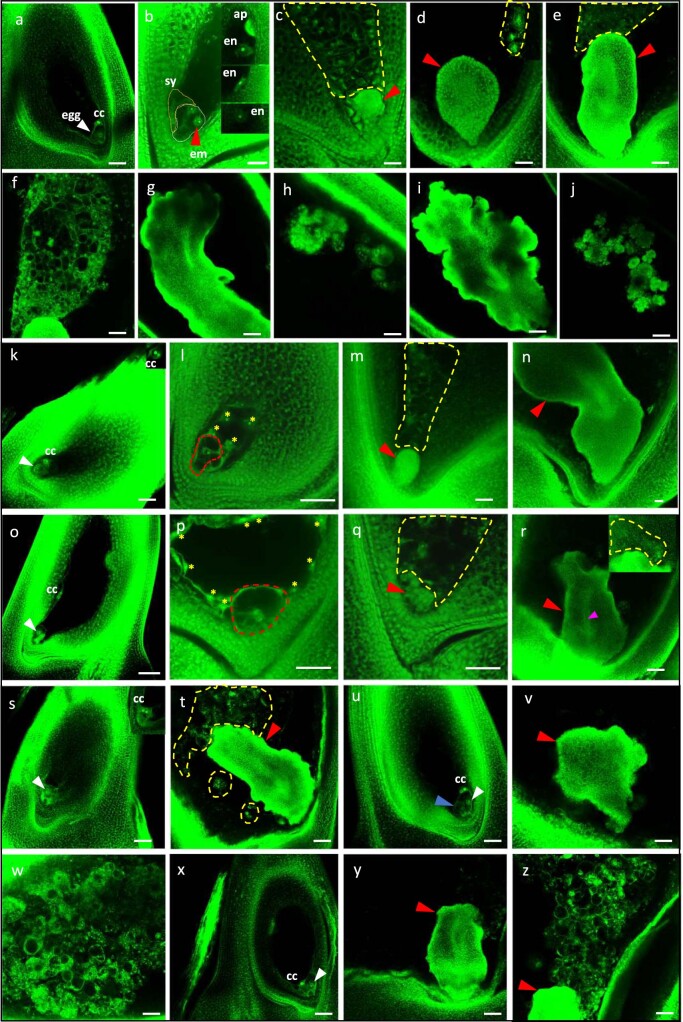
Asexual embryos and autonomous endosperm in different double mutants. **a**, Embryo sacs of WT segregants of *Osfie-double2* at 12 DPE, showing an egg (white arrowhead), and two central nuclei (cc) (n = 68). **b**, Asexual four celled pre-embryo (circled by white dashed line, with another two out of focus) with a possible degenerating synergid cell (circled by yellow dashed line) and autonomous endosperm nuclei (inserted) in *Osfie-double2* at 0 DEP. em: four-celled asexual embryo (red arrowhead); sy: synergid cell; en: autonomous endosperm; ap: antipodal (n = 1). **c**, Asexual globular embryos (red arrowhead) and cellularized endosperm (circled by yellow dashed line) in *Osfie-double2* at 7 DPE (n = 31 in c-f). **d**, Asexual globular embryos (red arrowhead) and endosperm (circled by yellow dashed line) in *Osfie-double2* at 7 DPE. **e**, Asexual embryos (red arrowhead) and endosperm (circled by yellow dashed line) in *Osfie-double2* at 7 DPE. **f**, Cellularized autonomous endosperm for the ovule in e. **g** & **h**, Elongated asexual embryos (g) and starchy endosperm (h) from ovules in *Osfie-double2* at 12 DPE (n = 8 in g-j). **i** & **j**, Elongated asexual embryos (i) and starchy endosperm (j) from ovules in *Osfie-double2* at 12 DPE. **k**, Embryo sac of an *Osfie1* homozygous segregant of *Osfie-double4* at 10 DPE, showing an egg (white arrowhead), and two central nuclei (cc) (n = 24). **l**, Autonomous endosperm in *Osfie-double4* at 0 DPE (egg circled by red dashed line and endosperm nuclei marked by yellow asterisks) (n = 19). **m**, Autonomous development in *Osfie-double4* at 5 DPE (red arrowhead for embryo and yellow circle for endosperm) (n = 5). **n**, Asexual embryo (red arrowhead) in *Osfie-double4* at 12 DPE (n = 3). Endosperm is absent. **o**, Embryo sacs showing no autonomous development in double homozygous segregants with *Osfie2-6* weak allele of *Osfie-double3* at 9 DPE (n = 41); white arrowhead for egg; cc for central cell nuclei. **p**, Autonomous development in *Osfie-double3* at 0 DPE (egg circled by red dots and endosperm nuclei marked by yellow asterisks) (n = 9). **q**, Autonomous development in *Osfie-double3* at 4 DPE (red arrowhead for embryo and yellow dashed line circle for endosperm) (n = 8). **r**, Autonomous development in *Osfie-double3* at 12 DPE (red arrowhead for embryo, yellow dashed line circle for endosperm, pink arrowhead for vascular structure) (n = 5). **s**, Embryo sacs showing an egg cell (white arrowhead) and central cell nuclei (cc) in *Osfie1* heterozygous segregants of (WT X *Osfie-double1*)F1 at 12 DPE (n = 58). **t**, Embryo sacs showing autonomous development in double heterozygous segregants of (WT X *Osfie-double1*)F1 at 12 DPE (red arrowhead for embryo and yellow dashed line circle for endosperm) (n = 4). **u**, Embryo sacs showing an egg cell (white arrowhead), synergid (blue arrowhead) and central cell nuclei (cc) in double heterozygous segregants with *Osfie2-6* weak allele of (WT X *Osfie-double3*)F1 at 12 DPE (n = 59). **v** & **w**, Embryo sacs showing autonomous development in double heterozygous segregants of (WT X *Osfie-double3*)F1 at 12 DPE (red arrowhead for embryo and endosperm in W) (n = 13). **x**, Embryo sacs showing an egg cell (white arrowhead) and central cell nuclei (cc) in *Osfie1* heterozygous segregants of (WT X *Osfie-double4*)F1 at 12 DPE (n = 64). **y** & **z**, Embryo sacs showing autonomous development in double heterozygous segregants of (WT X *Osfie-double4*)F1 at 12 DPE (red arrowhead for embryo) (n = 26). Bar = 50 μm.

The shrivelled seed formation in the *Osfie* double mutants is similar to that in the *Arabidopsis* *fie* mutant and prompted us to investigate autonomous endosperm formation in the double mutants. We examined emasculated ovules of *Osfie-double1* heterozygotes, with the *Osfie1* homozygous segregants as control at T_2_ and sampled the ovules at different stages for confocal microscopy (Supplementary Table 1). Autonomous endosperms were seen in ovules at a frequency of ~50% (26/57) at 0 d post emasculation (DPE) (Figs. 3c,d and 5a, and Supplementary Table 1), with some ovules showing slight elongation (Extended Data Fig. 2q), while the other half exhibited a wild-type morphology (Fig. 3a,b). Interestingly, ~10% (6/57) of ovules that had autonomous endosperm also had globular embryo-like structures (Figs. 3c and 5a, and Supplementary Table 1), with no signs of eggs and synergids. Other ovules that had autonomous endosperm but no embryo always contained intact eggs characterized by having large nuclei and vacuoles but no synergids (Fig. 3d). We speculated that synergids might be disintegrated by autonomous endosperm in a similar way in which early sexually derived endosperm fuses to one remaining synergid soon after fertilization^28^. Ovules at 1, 2 and 3 DPE showed similar autonomous development of endosperm in ~50% of the ovules and the frequency of these asexually derived embryos occurring gradually increased (Figs. 3e and 5a, and Supplementary Table 1). From 6 DPE, we started to observe a few ovules with asexual embryos without clear presence of autonomous endosperm. We reckoned that the endosperm might degrade in these ovules. Therefore, we defined the ovules with embryos and/or endosperm as autonomous seeds for scoring the frequency of autonomous development (Fig. 5a and Table 1). At 6, 9 and 14 DPE, ovules showing autonomous seed formation accounted for ~50% of the total emasculated ovules, with the ratios of asexual embryos being elevated (5/27 at 6 DPE, 17/39 at 9 DPE and 9/26 at 14 DPE; Supplementary Table 1 and Fig. 5a). Ovules at 9 DPE contained structures resembling embryos and/or cellularized endosperm in ~50% of the ovules examined (Figs. 3f and 5a, and Supplementary Table 1), suggesting that the later asexual embryos were probably derived from eggs with the possibility that the embryos in early emasculated ovules could originate from synergids. At day 14, the asexual embryo-like structures exhibited various morphologies, with some showing signs of vascular bundles (Fig. 3g and Supplementary Table 1). At maturity, the autonomous seeds contained arrested structures, with some being accompanied by abnormally developed starchy endosperm as shown by staining with an iodine and potassium-iodide solution (Fig. 3i). The ovules (*n* = 59) of *Osfie1* homozygous segregants did not display asexual embryo or autonomous endosperm formation after emasculation (Fig. 3j and Supplementary Table 1). We then pollinated WT with *Osfie-double1* at T_3_ and analysed the autonomous phenotype in the F_1_ plants. All emasculated double heterozygous segregants in the progeny of the cross exhibited the asexual embryos (4/14) and autonomous seed formation (7/14), while the *Osfie1* heterozygous segregants in the cross did not (0/58) (Supplementary Table 1 and Extended Data Fig. 3s,t).

**Extended Data Fig. 4:**
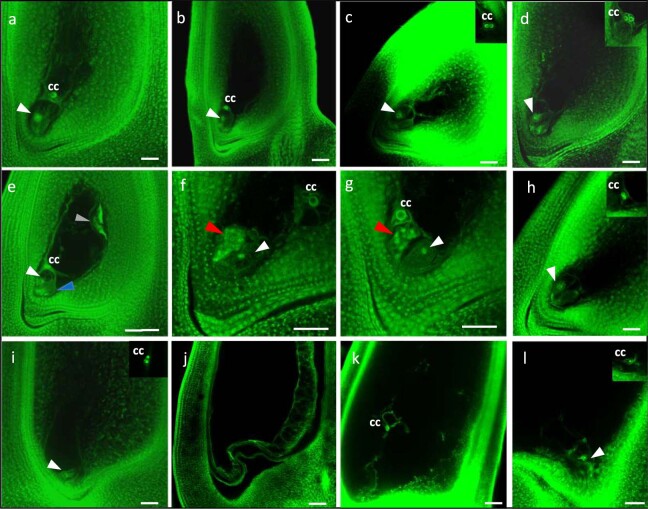
Emasculated ovules of WT, *Osfie1*, and *Osfie2*. **a**, Embryo sacs in Nipponbare at 15 DPE, showing an egg (white arrowhead), and two central nuclei (cc) (n = 49). **b**, Embryo sacs in Os*fie1-single1* at 15 DPE, showing an egg (white arrowhead), and two central nuclei (cc) (n = 7). **c**, Embryo sacs in Os*fie1-single2* at 10 DPE, showing an egg (white arrowhead), and two central nuclei (cc) (n = 5). **d**, Embryo sacs in Os*fie1-single3* at 12 DPE, showing an egg (white arrowhead), and two central nuclei (cc) (n = 24). **e**, WT-looking embryo sacs in *Osfie2-single2* at 0 DEP, showing an egg (white arrowhead), two synergids (one in focus by blue arrowhead, sy), two central nuclei (cc), and antipodal cells (grey arrowhead, ap) (n = 14). **f**, Asexual pre-embryo like structures (red arrowhead) at 8 DPE and a likely egg (white arrowhead) in *Osfie2-single2* (n = 2 in f and g). **g**, Asexual pre-embryo like structures (red arrowhead) at 8 DPE and a likely egg (white arrowhead) in *Osfie2-single2*. **h**, Embryo sacs of WT segregants in *Osfie2-single2* at 12 DPE, showing an egg (white arrowhead), and two central nuclei (cc) (n = 78). **i**, Embryo sacs of emasculated Nipponbare 4 days after auxin treatment, showing an egg (white arrowhead), and two central nuclei (cc) (n = 11). **j**, Embryo sacs of emasculated *fie1-single1* 14 days after auxin treatment (n = 10). **k**, Embryo sacs of emasculated *fie1-single1* 7 days after auxin treatment, showing two central nuclei (cc) (n = 10). **l**, Embryo sacs of emasculated Nipponbare 7 days after auxin treatment; central cell (insert), showing an egg (white arrowhead), and two central nuclei (cc) (n = 11) Bar = 50 μm.

We analysed three more independently isolated double mutant lines to further characterize asexual embryo formation and exclude the possibility that the phenotypes were caused by certain guide RNA (gRNA) combinations, off-targeting gRNA or tissue culture-induced effects. We examined ovules from *Osfie-double2* at 0 DPE to investigate the origins of asexual embryos. We found one ovule (1/56) showing a four-celled pre-embryo with a degenerating synergid and limited autonomous endosperm nuclei, while others are either WT or ovules with more advanced asexual globular embryos as shown in other lines (Extended Data Fig. 3b and Supplementary Table 1). This four-celled embryo is full of vacuoles and resembles a dividing zygote, suggesting that it originated from an egg^29^. Among the progeny of *Osfie-double2* at T_4_ and F_1_ of WT × *Osfie-double4* (T_3_) (Fig. 1c), the emasculated double heterozygous segregants exhibited autonomous seed formation in ovules (53/99 for *Osfie-double2*; 40/78 for *Osfie-double4*) and asexual embryos (39/74 for *Osfie-double2*; 26/78 for *Osfie-double4*), while the WT or *Osfie1* homozygous segregants did not (WT *n* = 68, *Osfie1* *n* = 64; Table 1, Extended Data Fig. 3a–j,x–z and Supplementary Table 1). Similarly, among the F_1_ progeny of WT × *Osfie-double3* (Fig. 1c), the autonomous phenotype only occurred in the ovules of double heteroallelic segregants (20/42 for autonomous seed formation; 13/42 for asexual embryo formation), while the ovules of the *fie1-3* and *Osfie2-6* double heterozygous segregants (*n* = 59) did not show autonomous phenotype as *Osfie2-6* is a weak allele (Extended Data Fig. 3u–w, Supplementary Table 1 and Table 1). By analysing the *Osfie-double3* and *4* at T_2_, we observed similar autonomous phenotypes in the ovules of double mutants carrying loss-of-function *Osfie2* alleles, while the control segregants did not show autonomous growth (Supplementary Table 1 and Extended Data Fig. 3k–r). We further emasculated three independently isolated *Osfie1* homozygotes and Nipponbare and found no asexual embryo or autonomous endosperm formation (Table 1, Supplementary Table 1 and Extended Data Fig. 4a–d). As all the mutants were generated by the same gene construct and were obtained by the same transformation process, the three independent *Osfie1* homozygous lines and the four independent double mutant lines should have borne the same chance of having or not having the asexual embryo phenotype if off-targeting or tissue culture-induced effects were responsible. However, only the four double mutant lines exhibited the phenotype but not the *Osfie1* lines, suggesting that the double mutations are probably responsible for the asexual embryo formation. Since all the 25 segregants, which did not carry loss-of-function *Osfie2* alleles, exhibited no asexual embryo formation in the progeny of the selfed or out-crossed double mutant lines (Supplementary Table 1), if the gRNA off-targeting or tissue culture effects had caused the phenotype, the probability of all the 25 segregants showing no asexual embryo formation would be close to zero (50% to the power of 25, assuming each segregant had 50% equal chance of inheriting the off-targeting-induced mutations). Therefore, it is highly unlikely that gRNA off-targeting or tissue culture effects were involved in inducing the asexual embryo formation in the double mutants.

**Extended Data Fig. 5:**
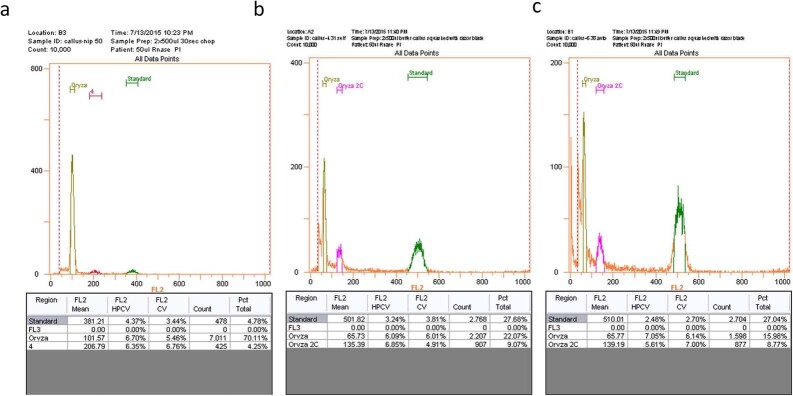
The flow cytometric DNA histograms showing the gating borders (bars above the Propidium iodide florescence intensity peak) and peaks of rice samples (olive and pink) and internal control *Bellis prennis* (green). **a**, Nipponbare callus showing a 2C (olive) and 4C (pink) peaks. **b**, Callus from aborted embryo of a self-pollinated seed showing 1C (olive) and 2C (pink) peaks. **c**, Callus from asexual embryo showing 1C (olive) and 2C (pink) peaks.

The gametophytic nature of the seed abortion and the autonomous seed formation, and the 1:1 segregation of two genotypes in the heterozygous double mutant lines, as in the *Arabidopsis* *fie* mutant, imply that meiosis must have occurred normally to give rise to the viable WT (or *Osfie1*) embryo sac and the *Osfie1/Osfie2* embryo sac (Fig. 2h). Therefore, the egg apparatus-derived asexual embryos are probably haploid. To test this, we were able to induce callus formation from 3 of ~100 asexual embryos with enough biomass for detection of DNA content by flow cytometry^30,31^. As expected, all the induced calli (*n* = 3) were haploid, while the DNA contents from WT leaves and calli induced from sexually derived embryos were diploid (Fig. 5b–e, Extended Data Fig. 5 and Supplementary Table 2). Given that the collapsed seeds of the double mutants under self-pollination morphologically resemble the autonomous seeds (Fig. 3h,i and Extended Data Fig. 2) and ~50% ovules of double mutants showed autonomous development at 0 DPE, we reasoned that ovules with the double mutations must have initiated autonomous development before pollen dehiscence and that the defective embryos were of asexual origin. We successfully induced 3 calli from ~100 abnormal embryos from self-pollinated ovules and found that these calli contained similar DNA contents as the asexual embryos (Fig. 5b–e). We further verified the maternal genotype of aborted embryos by pollinating *Osfie-double1* with an *indica* rice 9311. By analysing the genotypes of both viable and aborted seeds via sequencing of the PCR products amplified from a region containing single nucleotide polymorphisms (SNPs) at *OsYUCCA11* (Fig. 5f), we observed that the aborted embryos (*n* = 11) only showed the maternal SNP, while the viable embryos (*n* = 16) had two parental SNPs, suggesting that the aborted seeds were of asexual origin.

### Asexual pre-embryo-like structures in *Osfie2* mutants

**Fig. 4:**
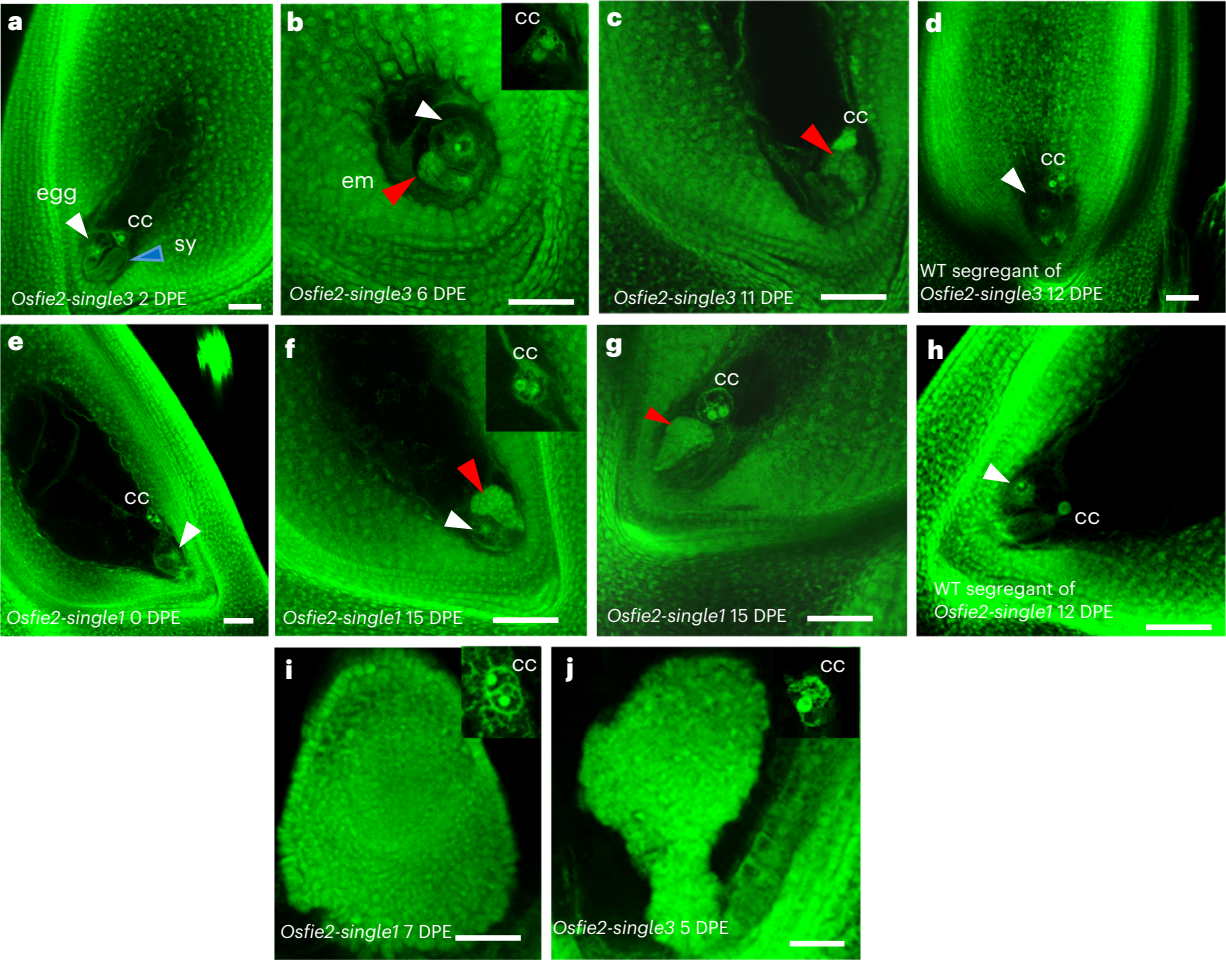
Asexual embryo development in two *Osfie2* single mutant lines. **a**, WT-looking embryo sacs in *Osfie2-single3* at 2 DPE, with an egg (white arrowhead), two synergids (one in focus by blue arrowhead) and two central nuclei (*n* = 25). **b**, Asexual pre-embryo-like structure (red arrowhead) in *Osfie2-single3* at 6 DPE, with an egg-like cell (white arrowhead) and central cell nuclei (insert) (*n* = 1). **c**, Asexual pre-embryo-like structures (red arrowhead) in *Osfie2-single3* at 11 DPE, with central cell nuclei and without an egg (*n* = 5). **d**, Embryo sacs of a WT segregant in *Osfie2-single3* at 12 DPE, showing an egg (white arrowhead) and two central nuclei (*n* = 18). **e**, WT-looking ovules of *Osfie2-single1* at 0 DPE, showing an egg (white arrowhead) and two central nuclei (*n* = 8). **f**, Asexual embryo-like structures (red arrowhead) in *Osfie2-single1* at 15 DPE, accompanied by an egg-like cell (white arrowhead) and two central cell nuclei (insert) (*n* = 14 in **f** and **g**). **g**, Asexual embryo-like structures (red arrowhead) in *Osfie2-single1* at 15 DPE, with central cell nuclei and without an egg. **h**, Embryo sacs of WT segregants of *Osfie2-single1* at 12 DPE, showing an egg (white arrowhead) and two central nuclei (*n* = 20). **i**, Emasculated ovules of *Osfie2-single1* treated with 2,4-d containing a proliferated asexual embryo-like structure at 7 DPE, with central cell nuclei (insert) (*n* = 3). **j**, Emasculated ovules of *Osfie2-single3* treated with 2,4-d containing a proliferated asexual embryo-like structure at 5 DPE, with central cell nuclei (insert) (*n* = 6). Scale bars, 50 μm.

These novel asexual embryo-like structures in the four independent *Osfie* double mutants prompted us to investigate whether this phenotype also occurred in the three independent *Osfie2* mutants. This would clarify whether the two *OsFIE* genes play a redundant role in modulating the autonomous phenotype. We emasculated all three independent *OsFIE2* +*/−* heterozygous lines at T_2_ and T_3_, using WT segregants as control (Supplementary Table 1). In *Osfie2-single3*, we found the embryo sacs from 2 to 4 DPE (*n* = 48) displaying a WT morphology (Fig. 4a), as in Nipponbare (Fig. 3a), and giving neither asexual embryo nor autonomous endosperm phenotypes. At 6 DPE, we observed one multicellular structure resembling an early embryo at the micropylar end of the emasculated ovule, accompanied by an egg-like cell characterized by having a large nucleus with clear nucleolus and vacuoles, two central cell nuclei and proliferated antipodal from 22 ovules (Fig. 4b and Supplementary Table 1). At 11 DPE, we observed more asexual pre-embryo-like structures (5/33), sometimes without an egg (Fig. 4c and Supplementary Table 1). At 12 DPE, we observed more asexual pre-embryo-like structures in ovules (5/23), while the WT segregants gave no such structures after emasculation (*n* = 72; Fig. 4d and Supplementary Table 1). At T_3_, 12 asexual pre-embryos were observed in 63 ovules from three heterozygous segregants (Supplementary Table 1). In *Osfie2-single1*, the embryo sacs from 0 to 6 DPE (*n* = 85) exhibited no asexual embryo formation but a WT morphology (Fig. 4e). At 9 DPE, we observed one asexual pre-embryo-like structure (1/8) at the micropylar end of the emasculated ovule. At 15 DPE, we observed more similar structures (8/34), with 6 more spotted without scoring the total ovules (Fig. 4f,g and Supplementary Table 1). At T_3_, 22 asexual pre-embryos were observed in 99 ovules from three heterozygous segregants. The ovules of WT segregants at T_2_ gave no asexual embryo formation after emasculation (*n* = 42) (Supplementary Table 1 and Fig. 4h). In *Osfie2-single2*, we observed similar asexual embryo-like structures with a similar frequency in the 8 and 12 DPE ovules (6/33) at T_2_ and 21 DPE ovules (5/22) at T_3_, while the ovules (*n* = 78) of WT segregants did not display asexual embryo formation (Extended Data Fig. 4e–h, Table 1 and Supplementary Table 1). We did not find any autonomous division of the central cell in all *Osfie2* mutants, in contrast to the previous finding of a low frequency of autonomous endosperm formation in *Osfie2* mutants^14^. The absence of asexual embryo formation in all 10 WT segregants of the selfed *Osfie2* heterozygous lines and the presence of asexual embryo formation in all the single *Osfie2* and double mutant lines support the idea that the single *Osfie2* mutation is responsible for the asexual embryo formation. The female gametophyte carrying the *Osfie2* single mutation could be fertilized and gave rise to normal-looking heterozygous plants (Fig. 2e), suggesting that these asexual embryos derived from the *Osfie2* embryo sacs are also haploid. We further scored the asexual pre-embryos of these lines from quality confocal images for the presence and absence of accompanying eggs and found that ~40% (28/71) were accompanied by egg-like cells, while others were not, indicating that these structures might well be derived from the eggs and synergids. The asexual embryo-like structures in the *Osfie2* mutants remained small compared with those in the double mutants and arrested without further development, suggesting that the autonomous endosperm in the double mutants facilitated the asexual embryo development.

Previous studies suggest that FIS PRC2 represses auxin production in the central cell, and fertilization brings in the paternally expressed auxin biosynthesis genes that trigger central cell division. Exogenous application of auxin 2,4-d induces autonomous endosperm development in *Arabidopsis*^16^. Emasculated florets were treated with a synthetic auxin (2,4-d) of different concentrations from 10 µm to 200 µm, but no endosperm or embryo formation was induced in wild type or *Osfie1*, while the pericarps elongated as expected (Extended Data Fig. 4i–l). Interestingly, by treating emasculated *Osfie2-single3 and Osfie2-single1*, we observed proliferated cell masses with early emergence at the micropylar ends of the emasculated ovules at a frequency of ~20% (9/50 for *Osfie2-single3* at 5 and 6 DPE, and 8/43 at 2–4 DPE; 11/60 at 15 DPE for *Osfie2-single1*) similar to the asexual pre-embryos in the *Osfie2* mutants without treatment (Supplementary Table 1 and Fig. 4i,j), suggesting that the external application of auxin facilitated the early onset and cell division of the asexual pre-embryos. However, these proliferated cell masses did not show any signs of differentiation.

### Transcriptomic analysis of asexual embryos and autonomous endosperm

**Extended Data Fig. 6:**
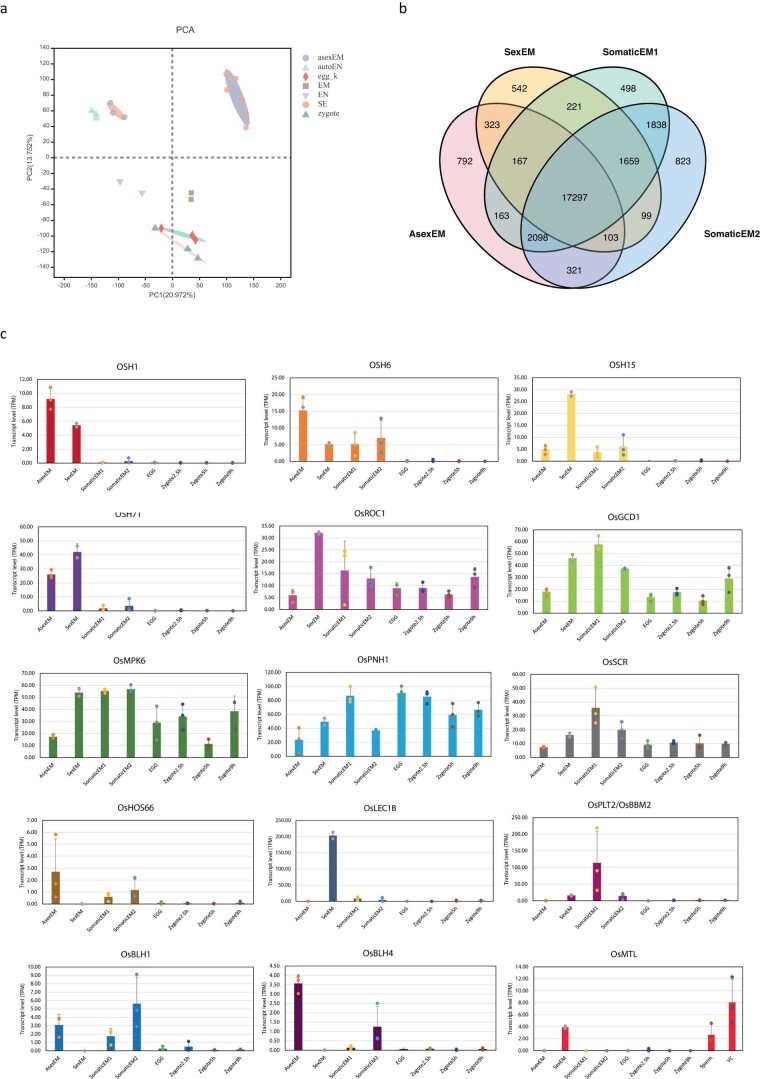
Transcriptomic analysis of asexual embryo and endosperm of *Osfie-double1*. **a**, Principal Component Analysis (PCA) analysis for the transcriptomic samples, asexEM (asexual embryo with triplicates), autoEN (autonomous endosperm with triplicates), EM (5 days sexual embryo), EN (5 days sexual endosperm), SE (Somatic embryo), egg, Zygote (zygote 9 hours post fertilization). **b**, Venn diagram showing overlapping of expressed genes in asexual (asex), sexual (sex) and somatic embryo (EM) transcriptomes. **c**, Expression of genes potentially involved in embryo pattern formation in various tissues, using transcriptome data (*OSH1*, *OSH6*, *OSH15*, *OSH71*, *OsROC1*, *OsGCD1*, *OsMPK6*, *OsPNH1*, *OsSCR, OsHOS66*, *OsLEC1B*, *OsPLT2/OsBBM2*, *OsBLH1*, *OsBLH4* and *OsMTL*; VC: Vegetative cell). Data are presented as mean values ± s.d. of 3 (AsexEM, somaticEM1, somticEM2, Egg, zygote2.5h, zygote5h, zygote9h,) and 2 (SexEM) biological replicates. Source data

To understand how the FIE function represses autonomous development, we isolated RNAs from the autonomous endosperm and asexual embryos from selfed *Osfie-double1* with three biological repeats to generate transcriptome data using the Illumina platform (Supplementary Table 3). We also included the transcriptomic datasets from the sexually derived embryo and endosperm in our previous study^32^, the egg, the zygotes of different stages^3,33^ and somatic embryos^34^. A principal component analysis (PCA) of these transcriptomes showed that autonomous endosperm and asexual embryos are grouped closer to each other than to other tissues including embryos, somatic embryos, endosperm, egg and zygotes (Extended Data Fig. 6a), suggesting that the maternal origin and/or severely perturbed development of these autonomous structures with compromised H3K27me^3^ might affect similar target genes and contribute to the close positioning of these two sets of transcriptomic data.

**Fig. 5:**
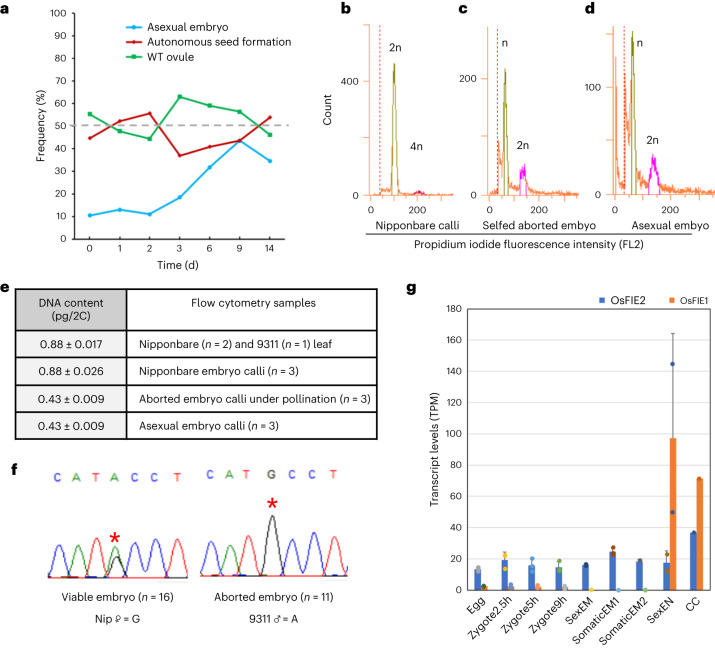
Frequency of autonomous seed formation, ploidy level of asexual embryos, and maternal origin of aborted embryos in *Osfie-double1* and *OsFIE* gene expression. **a**, Frequency of asexual embryo and autonomous seed formation at different days post emasculation (extracted from Supplementary Table 1). **b**–**d**, Flow cytometric DNA histograms for ploidy levels of Nipponbare (Nip) calli (**b**), calli from aborted embryo of self-pollinated seeds (**c**) and asexual embryo (**d**) of *Osfie-double1*; the gating borders and peaks for internal standards (*Bellis perennis*) are shown in Extended Data Fig. 5. The positions of peaks for the internal control were slightly different between the histograms (Extended Data Fig. 5) and would not affect measurement of the absolute DNA contents for each sample (Supplementary Table 2). **e**, Cellular DNA content of aborted embryos under pollination, asexual embryos and wild-type tissues by flow cytometry (Supplementary Table 2). **f**, Sanger sequencing of *OsYUCCA11* PCR fragments amplified from embryos of the double heterozygote (in Nip background) pollinated with 9311 (*indica*), showing that viable embryos were hybrids with double peaks (left, *n* = 16) and aborted embryos are maternal with a single Nip peak (right, *n* = 11); red asterisks indicate the SNPs between Nip and 9311. **g**, Expression of *OsFIE1* and *OsFIE2* in different tissues using publicly available transcriptomic data. CC, central cell. Data are presented as mean ± s.d. of 3 (egg, zygote2.5h, zygote5h, zygote9h, somaticEM1, somaticEM2), 2 (SexEM and SexEN) and 1 (CC) biological replicate. Source data

**Fig. 6:**
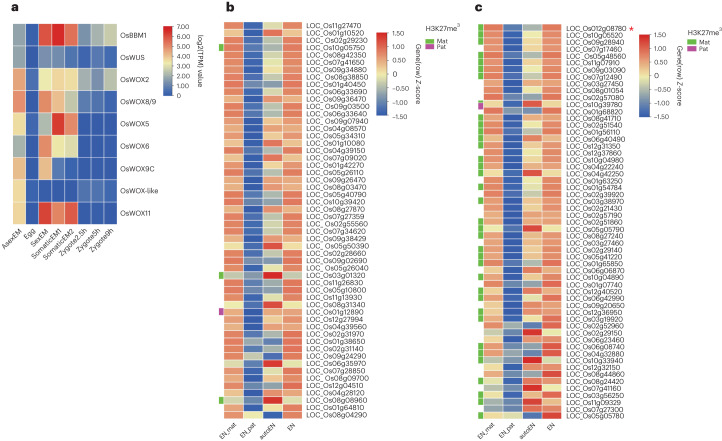
Gene activation in asexual embryo and autonomous endosperm, and PEGs enriched with parentally biased H3K27me^3^ marks. **a**, Heat map showing expression levels of *BBM1* and *WUS* family genes in asexual embryo (asexEM) egg^3,33^, embryo^32^, somatic embryo (somaticEM;^34^) and zygote^3,33^ (Supplementary Table 6). **b**, Heat map showing the expression levels of paternal alleles (EN_pat) and maternal alleles (EN_mat) of MEGs in hybrid endosperm (Supplementary Table 13; ref. ^32^) and expression levels of the same genes in autonomous endosperm (auto_EN; Supplementary Table 13). Lane EN shows the total expression level of combined parental alleles of MEGs in hybrid endosperm. Small bars on the left (in **b** and **c**) indicate which genes are marked with H3K27me^3^ at the maternal allele (green bars) or the paternal allele (purple bar), or have no marks detected (no bars) (Supplementary Table 15). **c**, Heat map showing the expression levels of paternal alleles (EN_pat) and maternal alleles (EN_mat) of PEGs in hybrid endosperm (Supplementary Table 12; ref. ^32^), and expression levels of the same genes in autonomous endosperm (auto_EN; Supplementary Table 12). Lane EN shows the expression levels of combined parental alleles of PEGs in hybrid endosperm. Red asterisk represents *OsYUCCA11*. Source data

**Extended Data Fig. 7:**
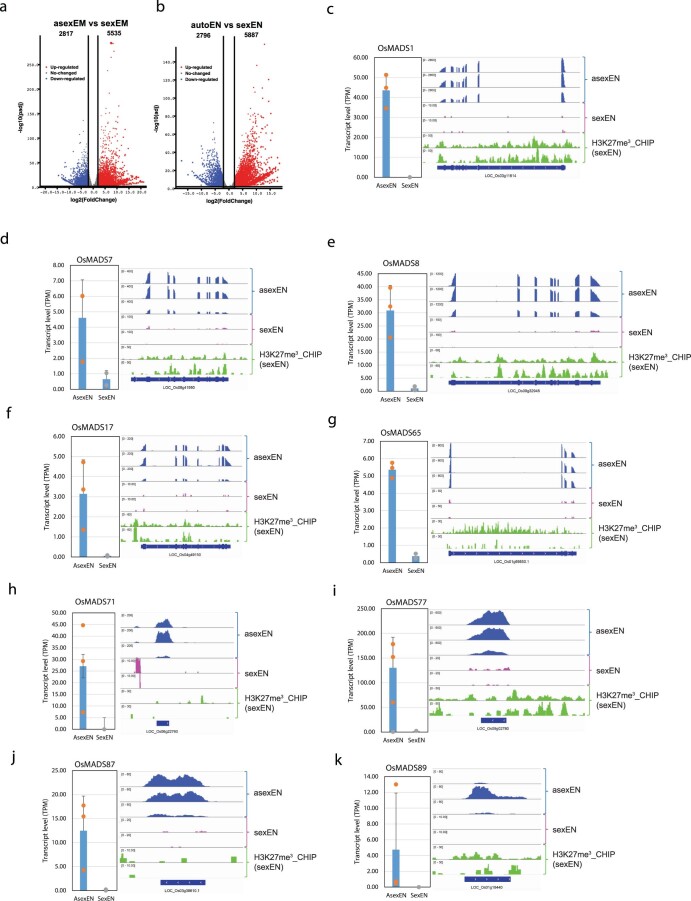
Differential gene expression, and visualization of gene expression and H3K27me^3^ enrichment of type I MADS-box genes. **a**, Volcano plot showing genome-wide differential gene expression between asexual (asexEM) and sexual embryo (sexEM) (Supplementary Table 4, using DESeq2^76^ R package). X axis showing Log2 values of fold change. Y axis showing the statistical significance (-Log10(padj)) of the expression change and the padj was selected as 0.05. The grey dots represent genes with expression of no more than 4 times fold change. Red dots represent genes with 4 times higher expression (n = 5535), while blue dots represent 4 times lower expression in asexual embryo (n = 2817). **b**, Volcano plot showing genome-wide differential gene expression between autonomous (asexEn) and sexual endosperm (sexEN) (Supplementary Table 7; using DESeq2^76^ R package). X axis showing Log2 values of fold change. Y axis showing the statistical significance (-Log10(padj)) of the expression change and the padj was selected as 0.05. The grey dots represent genes with expression of no more than 4 times fold change. Red dots represent genes with 4 times higher expression (n = 5887) while blue dots represent 4 times lower expression in autonomous endosperm (n = 2796). **c–k**, Type I MADS-box genes (*OsMADS1*, *OsMADS7, OsMADS8*, *OsMADS17*, *OsMADS65*, *OsMADS71*, *OsMADS77*, *OsMADS87* and *OsMADS89*) with higher expression in autonomous endosperm than in sexual endosperm showing enrichment of H3K27me^3^ in sexual endosperm. Data are presented as mean values ± s.d. of 3 (AsexEN) and 2 (SexEN) biological replicates shown. Source data

We identified over 17,000 commonly expressed genes in the transcriptomes of asexual, sexual and somatic cell-induced embryos (Extended Data Fig. 6b), which include genes that modulate the early pattern formation of the somatic embryo in rice^3,34–44^. Those genes are expressed at a comparable level to that in the sexual embryo (Extended Data Fig. 6c), suggesting that these asexual structures had acquired some degree of the characteristics of a sexual embryo at the molecular level. We identified 8,352 differentially expressed genes, including 5,535 upregulated and 2,817 downregulated genes between asexual embryos and sexual embryos (Supplementary Table 4 and Extended Data Fig. 7a). Gene Ontology (GO) term enrichment analysis of the differentially expressed genes in asexual embryos showed that they comprised diverse molecular functions and biological processes, consistent with the extensive disruption of the development and differentiation of the asexual embryos (Supplementary Table 5). We then focused on genes with possible functions in embryogenesis. The genes with de novo expression in zygote compared with egg may be involved in the initiation of embryogenesis^3,33^, among which some are male genome-expressed genes upon fertilization, while the female alleles in the egg and zygote are silenced. We asked how many of these genes with de novo expression in zygote are also expressed in the asexual embryo. We also extended the comparison to other *BBM* and *WUSCHEL* family members which are not included in the list of refs. ^3,33^. We found 36 genes activated in the asexual embryo (Supplementary Table 6), including 1 out of 4 *BBM*-like and 8 out of 11 *WUS* family genes, suggesting that the rice *FIE* genes may directly or indirectly repress expression of these genes in the egg apparatus and that they became activated without involvement of the paternal genome due to the mutations at the *FIE* loci. Among these genes with known embryogenic functions in plants are transcription factors, *OsBBM1* (LOC_Os11919060) and *WUS-LIKE HOMEOBOX GENES*^5–7,45^ (Fig. 6a).

Similarly, GO-term enrichment analysis on the differentially expressed genes (Supplementary Tables 7 and 8, and Extended Data Fig. 7b) between autonomous and sexual endosperm indicated that a wide range of molecular functions and biological processes of the autonomous endosperm had been altered (Supplementary Table 8). As auxin production and signalling in the primary endosperm are characteristic events after fertilization, we further analysed the expression of genes that are involved in auxin biosynthesis, transportation and signalling in autonomous endosperm (Supplementary Table 9). Most genes are expressed in autonomous endosperm, with *YUCCA* genes being expressed at a lower level than in sexual endosperm, indicating a possible low activity of auxin biosynthesis in the autonomous endosperm. The staining with an iodine and potassium-iodide solution of starch granules in autonomous endosperm prompted us to analyse the activity of genes involved in starch biosynthesis (Supplementary Table 10). Most of the starch biosynthesis genes have lower expression in autonomous endosperm than in sexual endosperm, consistent with the poor accumulation of starch in autonomous seeds, and this might be due to the lower gene dosage in autonomous endosperm. In rice, type I MADS-box transcription factor genes have been speculated to be involved in endosperm cellularization as is the case for the counterpart *AGL62* in *Arabidopsis*. We found that *OsMADS77* and *87* and several other MADS-box genes are activated in the autonomous endosperm as observed in *Osemf2a*^15,46^ (Supplementary Table 11 and Extended Data Fig. 7c–k).

**Extended Data Fig. 8:**
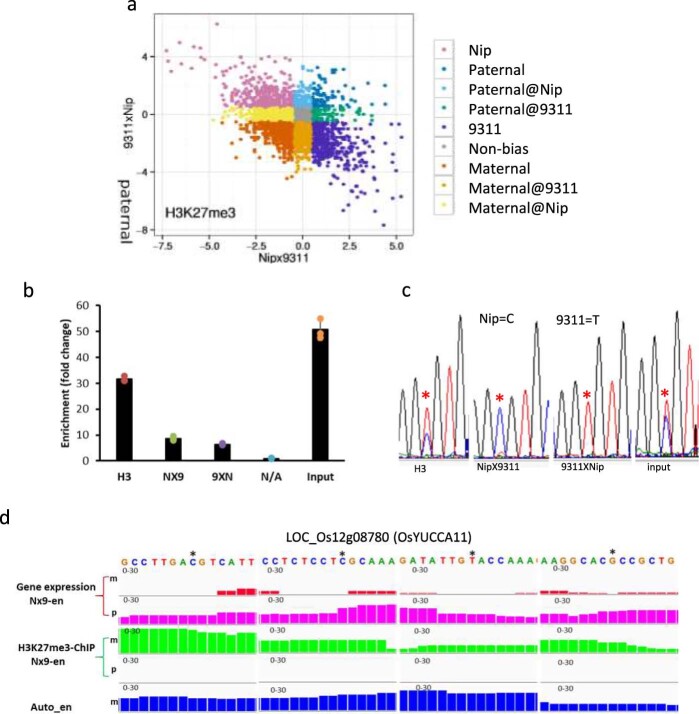
Chromatin immunoprecipitation assay with anti-body against H3K27me^3^ followed by deep sequencing (ChIP-seq) to identify H3K27me^3^ marked regions in the maternal or paternal genomes of endosperm from the reciprocal crosses between Nipponbare (Nip) and 9311. **a**, Dot plot indicating the parental bias status of H3K27me^3^ -enriched peaks based on the parental SNP counts in each of the reciprocal crosses, Nipx9311 and 9311xNip. Each dot represents a peak. Zero indicates no bias. Negative indicates H3K27me^3^ enrichment biased to maternal alleles. Positive indicates H3K27me^3^ enrichment biased to paternal alleles. Nip: biased to Nip allele in both crosses (436 peaks); Paternal: biased to paternal parents in both crosses (94 peaks); Paternal@Nip: biased to Nip in 9311xNip but no bias in Nipx9311 (136 peaks); paternal@9311: biased to 9311 in Nipx9311 but no bias in 9311xNip (140 peaks); 9311: biased to 9311 in both crosses (547 peaks); non-bias: no significant bias in both crosses (cutoff < 2^0.5) (577 peaks); Maternal: biased to maternal parents in both crosses (3602 peaks); Maternal@9311: biased to 9311 in 9311xNip but no bias in Nipx9311 (1454 peaks); maternal@Nip: biased to Nip in Nipx9311 but no bias in 9311xNip (1283 peaks). **b**, qPCR confirmation of H3K27me^3^ enrichment at *OsYUCCA11* locus using ChIP DNA prepared from hybrid endosperm harvested from the reciprocal crosses between Nip and 9311 six days after pollination. H3: ChIP performed with antibody against histone 3 in 9311xNip; NX9: ChIP performed with antibody against H3K27me^3^ in Nipx9311; 9XN: ChIP performed with antibody against H3K27me3 in 9311xNip; Input: input DNA in 9311xNip. Data are presented as mean values ± s.d. of 3 biological replicates. **c**, Sanger sequencing of the PCR products amplified on the above ChIP DNA and input DNA showing DNA enriched by antibody against histone 3 and input DNA have both parental SNPs, while the DNA enriched by antibody against H3K27me^3^ on the Nipx9311 or 9311XNip endosperm only gave the maternal SNPs. Asterisks indicate the SNPs. Similar experiments were performed for other PEGs and results are shown in Extended Data Fig. 9a. **d**, Transcription and H3K27me^3^ enrichment around 4 SNPs at *OsYUCCA11* locus for the NipX9311 endosperm, and transcription for the autonomous endosperm. For gene expression, m represents maternal normalized reads; p represents paternal normalized reads. For H3K27me^3^ ChIP, m represents maternal normalized reads; p represents paternal normalized reads. For autonomous endosperm, expression is shown by the normalized reads. More examples of other PEGs are in Extended Data Fig. 9. Source data

**Extended Data Fig. 9:**
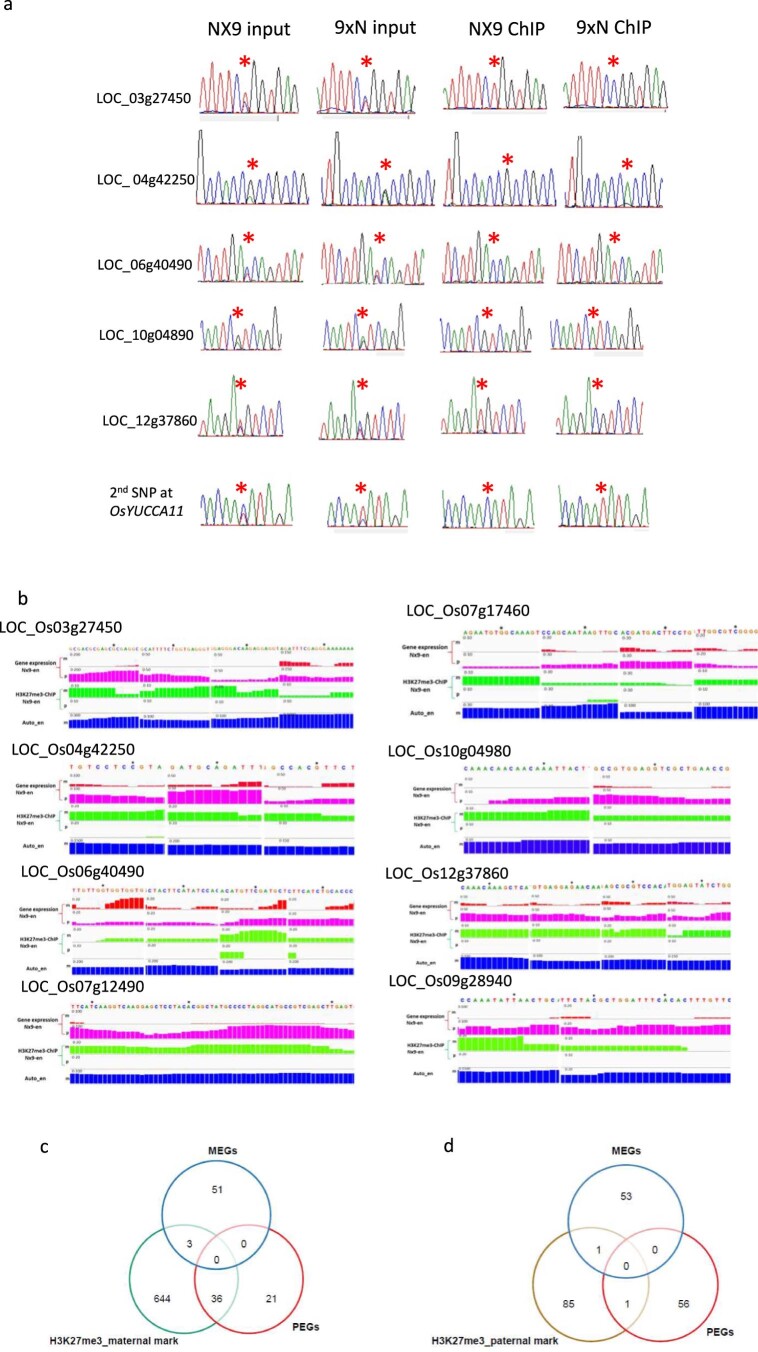
Sanger sequencing of PCR products amplified on H3K27me^3^ enriched DNA, and visualization of transcription and H3K27me^3^ enrichment for selected PEGs. **a**, Sanger sequencing of the PCR products amplified on the ChIP DNA and input for selected PEGs, showing input DNA samples have both parental SNPs (asterisks), while the DNA samples enriched by antibody against H3K27me^3^ on the Nipx9311 or 9311XNip endosperm only gave the maternal SNPs (asterisks). **b**, Transcription and H3K27me^3^ enrichment around 4 SNPs at selective PEGs for the NipX9311 endosperm, and transcription for the autonomous endosperm. For gene expression, m represents maternal normalized reads; p represents paternal normalized reads. For H3K27me^3^ ChIP, m represents maternal normalized reads; p represents paternal normalized reads. For autonomous endosperm, expression is shown by the normalized reads. **c**, Venn diagram showing genes marked with maternal H3k27me^3^ overlapping with imprinted genes. **d**, Venn diagram showing genes marked with paternal H3k27me^3^ overlapping with imprinted genes.

Endosperm is characterized by expression of maternally or paternally expressed imprinted genes. De-repression of paternally expressed imprinted genes *YUCCA10* and *TAR1* in auxin biosynthesis is thought to trigger autonomous endosperm development in *Arabidopsis* PRC2 *fis-*class mutants^18,19,32,47–50^. We compared the relative expression levels of all the maternally expressed imprinted genes (MEGs)^32^ between autonomous endosperm and sexual endosperm and found that most of the MEGs are expressed in autonomous endosperm (Fig. 6b and Supplementary Table 13). Interestingly, 100% (57/57) of the PEGs^32^ were also expressed in the autonomous endosperm, showing comparable levels to those in sexual endosperm (Fig. 6c and Supplementary Table 12). As the autonomous endosperm is maternally derived, we conclude that loss of FIE function activates the maternal alleles of the PEG loci. It is worth noting that the PEG *OsYUCCA11* was maternally activated in the autonomous endosperm (Fig. 6c and Supplementary Table 12). We used chromatin immunoprecipitation (ChIP-seq) with antibody against H3K27me^3^, followed by deep sequencing to identify H3K27me^3^-marked regions in the maternal or paternal genomes of endosperm from the reciprocal crosses between Nip and 9311 (Supplementary Method). The ChIP assay result suggests that the PRC2-modulated H3K27me^3^ targeted the maternal alleles of PEGs in endosperm (Fig. 6c, Supplementary Tables 14–17, Extended Data Figs. 8 and 9, and Supplementary Method), consistent with the idea that PEGs are activated in the autonomous endosperm^16^.

We then investigated the gene expression patterns of *OsFIE1* and *OsFIE2* by analysing transcriptomic data in egg, zygote, embryo, somatic embryo, endosperm^3,32–34^ and central cell (an unpublished transcriptome deposited in NCBI (DRR000623)). *OsFIE1* is relatively highly expressed in endosperm and central cell but very lowly expressed in egg and zygote (Fig. 5g), while *OsFIE2* is ubiquitously expressed in all tissues, consistent with the mutant phenotypes that *OsFIE1* and *OsFIE2* redundantly repress central cell division and regulate endosperm development, while OsFIE2 functions independently in the embryo to repress egg apparatus division.

## Discussion

Our study uncovers a novel role for the PRC2 component OsFIE2 in suppressing the autonomous division of the egg apparatus, in addition to the expected role of OsFIE1 and OsFIE2 in suppressing central cell division before fertilization in rice. The asexual pre-embryo-like structures in *Osfie2* mutants started to emerge at the micropylar end of emasculated ovules ~1 week after emasculation, with higher frequency of these structures being observed at later stages. Some of the asexual pre-embryos co-located with egg-like cells, while others existed without accompanying eggs, suggesting that asexual embryo formation might be derived from synergids and eggs. Further study is required to characterize the exact cell lineage of these asexual embryos. However, the analysis of the asexual embryo formation phenotypes in the *Osfie1Osfie2* double mutants suggests that asexual embryos are mainly egg derived. In the early ovules with autonomous endosperm, only a small portion of ovules contained asexual globular embryos, with the rest containing egg-like cells without accompanying synergids. The synergids might be disintegrated by autonomous endosperm in a way similar to the typical fusing of the early sexually derived endosperm with one remaining synergid after fertilization^28^. This allowed the egg to become the major cell type to develop into parthenogenetic embryos in the ovules of later stages. Rarely, a four-celled pre-embryo resembling a dividing zygote was observed, coexisting with a synergid and endosperm nuclei, further supporting the egg origins of most asexual embryos. We reasoned that the lack of autonomous endosperm formation in the *Osfie2* mutants might have allowed the synergids to survive without being consumed by endosperm^28^ and facilitated the formation of synergid-derived embryos. However, there is a possibility that the asexual embryos in early ovules might also be synergid derived in the double mutants as those in *Osfie2* mutants—a possibility requiring further investigation.

In contrast to the late emergence, low penetrance and early arrest of asexual pre-embryos in *Osfie2*, the earlier onset, higher frequency and better development of the asexual embryos in the double mutants suggest that the autonomous endosperm facilitates the asexual embryo development of the *Osfie2*-activated egg apparatus potentially by providing signals or nutrients. This is supported by the observation of starch granule formation and expression of genes that are involved in auxin biosynthesis, transportation, signalling and starch biosynthesis in autonomous endosperm. The asexual pre-embryo structures emerged earlier and grew into bigger cell masses in the *Osfie2* mutants when the emasculated ovules were treated with synthetic auxin 2,4-d, indicating that auxin may be one of the important signals to support embryo growth. A similar phenomenon has been observed in *Arabidopsis* where autonomous endosperm derived from the PRC2 *fis1/mea* mutant was able to support haploid embryo development^51^.

Our finding contrasts with a similar study where single knockout of *OsFIE2* and double knockout of the two *FIE* genes both caused autonomous endosperm formation at a very low frequency without asexual embryo formation phenotype^14^. In our study, the double linked mutations at both *OsFIE* loci induced very high penetrance of asexual embryo formation and autonomous endosperm phenotype, while the *Osfie2* single mutations caused asexual pre-embryo-like structures at a lower frequency. We used the same vector containing three guides, with two guides targeting the two *OsFIE* genes and one common guide targeting both genes to generate all mutants in the same transformation process. Three independently isolated *Osfie1* homozygotes, two induced by the specific *OsFIE1* guide and one by the common guide, did not show any autonomous phenotypes, while the three independent *Osfie2* heterozygotes by the common guide and the four double mutants by different guide combinations exhibited asexual embryo formation, with the control segregants displaying none of the phenotypes. It is unlikely that the phenotype was caused by gRNA off-targeting or tissue culture effects during transformation, as each of the 10 analysed lines should have borne the same chance to have or not have the phenotype if the *Osfie2* mutations were not responsible for the phenotype. The fact that there was no asexual embryo formation in the segregants lacking the *Osfie2* loss-of-function mutations in the progeny of the *Osfie2* or double mutant lines further support the idea that the *Osfie2* mutations caused the asexual embryo formation. The possible reason for the discrepancy between the two studies is that the mutants were generated in different genetic backgrounds where unknown modifiers may exist to suppress the autonomous phenotype^13,14^. The low penetrance of the autonomous endosperm phenotype might obscure the observation of the embryo phenotype in ref. ^14^, as we showed that the high penetrance of the autonomous endosperm facilitates the prevalence of the asexual embryos. In *Arabidopsis*, a likely parthenogenetic pre-embryo phenotype has been observed in the mutant of the non-canonical PRC2 member *AtMSI1*; however, this phenotype has not been convincingly demonstrated except for the autonomous endosperm phenotypes in other PRC2 *fis-*class mutants^10,52^. It remains to be investigated whether asexual embryo formation triggered by the loss of the PRC2 only occurs in certain species or even only exists in certain genetic backgrounds in rice. It is also tempting to revisit whether the egg in the *Arabidopsis* *fie* mutant would undergo limited division as in the *Atmsi1* mutant. Similar to the findings in the moss *Physcomitrella patens* that mutations in PRC2 genes result in fertilization-independent production of a sporophyte-like body on side branches of the gametophytic protonema filaments^53,54^, the formation of asexual embryos in the rice mutants suggests that the PRC2 complex may function as a universal mechanism via H3K27me^3^ to maintain reproductive integrity.

The transcriptomic data were generated from tissues in which the development programme was severely perturbed by the loss-of-function mutations of the essential epigenetic modification genes. Therefore, the results of the transcriptomic analysis should be interpreted with caution, especially when compared with transcriptomes from sexually derived embryos or endosperm. Nevertheless, we found that the rice *BBM* and *WUS* homologues which are silenced in egg^3,4^ are expressed in the asexual embryos, suggesting that these genes must be activated at some stages during asexual embryo formation without the involvement of male genome. Ectopic expression of the *BBM* and *WUS* genes in *Arabidopsis*, brassicas and cereals has been shown to promote somatic embryogenesis and shoot regeneration from tissue culture, suggesting that they act as pluripotency factors^55–57^. In rice, *OsBBM1*, *OsBBM2* and *OsWOX8/9* are initially male genome-expressed in the early zygote and ectopic expression of *OsBBM1* in egg triggers parthenogenesis, suggesting that the requirement for fertilization in embryogenesis is mediated by paternal genome transmission of pluripotency factors^3,4^. We propose that *OsBBM1* and other pluripotency factors, such as *WUS* homologues, are repressed by the PRC2 complex directly or indirectly in the egg apparatus until fertilization, which brings the male genome-expressed pluripotency factors. Loss of the FIE function leads to de-repression of the pluripotency factors via the loss of the H3K27me^3^, which triggers asexual embryo formation.

Similarly, maternal activation of the known PEGs in autonomous endosperm indicates that the maternal alleles of those PEGs were de-repressed by the *fie* mutations. The expression of both MEGs and PEGs in the autonomous endosperm suggests that the *fie*-activated central cells may have sufficient stimuli for autonomous division as the *fie*-activated egg apparatus. The repressed state of the central cell due to PRC2-mediated H3K27me^3^ can be overridden by the introduction of the active paternal alleles of PEGs with fertilization^24^, as occurs in the zygote where fertilization introduces the male genome-expressed pluripotency factors (such as *OsBBM1*) and these paternally derived PEG products act as pluripotency factors (including auxin biosynthesis genes^24^) to trigger endosperm formation. This suggests that the embryo and endosperm progenitor cells convergently adopt PRC2 to suppress asexual embryo and autonomous endosperm formation possibly through silencing of maternal alleles of male genome-expressed genes before fertilization. Our study sheds light on the interplay between epigenetic regulation and fertilization to ensure proper seed development. The mechanism of FIS-PRC2 repression of asexual embryo and autonomous endosperm formation and the high penetrance of the autonomous phenotype in the mutant may help to induce haploids for speed breeding^58^ or be harnessed to engineer synthetic apomictic crops—an emerging revolutionary tool for fixing heterosis and enhancing yield^4,59–62^.

## Methods

### Generation of CRISPR/Cas9-edited mutants

The rice genome has two *FIE* homologues (LOC_Os08g04270 and LOC_Os08g04290) that are closely linked (Fig. 1a). The CRISPR/Cas9 editing method was used for mutant generation as previously described^63^. We cloned three single guide RNA (sgRNA) sequences in the transformation binary vector. The target seed sequences (~20 bp) of the three sgRNAs were selected from the *OsFIE1* (LOC_Os08g04290) and *OsFIE2* (LOC_Os08g04270) coding regions, with two specifics for *OsFIE1* (g3: GTCACCGACACGAAGTACT) and for *OsFIE2* (g1: TCGTTCTACACACTGAGTT), respectively, and the third one targeting both genes (g2: CTCATCATTTCTGCAAGCA) (Fig. 1a). This would maximize the chance of generating mutations respectively and simultaneously at both closely linked loci. The rice small nuclear promoters OsU3, OsU6a and OsU6b were used to drive g1, g2 and g3 sgRNA, respectively, and the DNA for the three sgRNA expression were synthesized by IDT, with Type II restriction enzyme Bsa I sites attached (Supplementary Table 18). These synthesized sequences were inserted into the binary vector pYLCRISPR/Cas9-MH using the GoldenGate strategy, with Type II restriction enzyme Bsa I for digestion and T4 DNA ligase for ligation^63^.

The calli were induced from mature rice seeds (*Oryza sativa* ssp. *japonica* cv. Nipponbare) and transformation was performed by using *Agrobacterium tumefaciens* strain GV3101. Positive transformed calli were screened by hygromycin, and then used to regenerate transgenic plants^64^.

### Growth condition and mutation detection

The wild type of rice (*O. sativa* ssp. *japonica* cv. Nipponbare) and transgenic plants were grown in a glasshouse at 28 °C during the day and at 20 °C during the night under natural light. Gene-specific primers were designed to identify the mutations around target sites. Four sets of primer pairs flanking the target sequences were used to amplify the DNA isolated from transgenic plants for mutation detection. They are OsFIE2-T1-F: ACCTACAGCTGCCTCAAGGA and OsFIE2-T1-R: TATCAGCCACGTAGCAAGCA for target 1 at OsFIE2, OsFIE2-T2-F: GGTGGAAGATGTAGAACCTAGTGG and OsFIE2-T2-R: ATCCTATGCAATGCCATGTGAAA for target 2 at OsFIE2, OsFIE1-T2-F: TGTGGTTTCAGTGGGTCTTTAGC and OsFIE1-T2-R: TAAGATCCCTGTCTGCACATTCC for target 2 at OsFIE1, and OsFIE1-T3-F: CTGTGGAATGTGCAGACAGGGATC and OsFIE1-T3-R: GTGACATCAGAAGCTGGATGAGT for target 3 at OsFIE1 (Supplementary Table 18). The PCR products were directly used for Sanger sequencing and mutations were deduced from the sequencing traces. The transgenic plants, or transgene-free plants, were used for DNA extraction (Qiagen DNeasy Plant Pro Kit) and PCR amplified with above primers for Sanger sequencing to score the genotype.

### Emasculation and microscopy

Florets predicted to flower within 1–2 d were used for emasculation after removing other younger florets and opened florets. The emasculation was performed by removing the un-opened stamens after cutting off the top end of florets during late afternoon. The treated panicles were protected in paper bags to avoid being outpollinated.

Emasculated florets or pollinated florets were immediately fixed in freshly prepared FAA (formaldehyde:acetic acid:glycerol:alcohol at 5:6:5:50 + 34% distilled water)^65^ for at least 24 h, then washed with 50% ethanol and stored in 70% ethanol at 4 °C. The samples were hydrated sequentially in 50% ethanol, 30% ethanol and distilled water for 30 min at each stage. Hydrated caryopses were pre-treated in 2% aluminum potassium sulfate for 20 min and stained with 10 mg l^−1^ eosin B solution for 12 h at room temperature. The samples were post-treated in 2% aluminum potassium sulfate for 20 min, rinsed in distilled water three times and dehydrated in a series of ethanol solutions (30%, 50%, 70%, 90%, 100%). The samples were transferred to 50% ethanol:methyl salicylate for 2 h and then cleared in pure methyl salicylate for at least 2 h before confocal microscopy imaging. Images were collected using a Leica SP8 laser scanning confocal microscope (Leica) equipped with ×10 (NA = 0.3), ×20 (NA = 0.75) and ×40 (NA = 1.1) water immersion objectives. Excitation wavelength was 543 nm and an emission range of 510–650 nm^66^ was collected with a PMT detector at a pixel resolution of 2,048 × 2,048. All images were collected and pseudo-coloured with the Green LUT using Leica Application Suite v.3.5 (LASX, Leica Microsystems). The autonomous phenotypes were scored by the presence of asexual embryos and/or autonomous endosperm in the emasculated ovules of the analysed *Osfie* mutants to avoid the occasional pseudo-parthenocarpy in emasculated ovules. The identity of the egg cell was determined by the characteristic presence of a large nucleus with a large nucleolus and the formation of vacuoles, while synergids were identified by being denser, having a smaller nucleus and the presence or absence of a smaller nucleolus.

For starch granule detection, the autonomous seeds were stained using an iodine and potassium-iodide solution^67^ (2 mg I_2_, 20 mg KI ml^−1^). Images were collected using a Leica m205c dissecting microscope equipped with a ×0.63 objective and a Leica IC90e digital camera (Leica).

For cryo-sectioning, developing seeds were cut in half and fixed in FAA for 48 h. Fixed seeds were embedded in OCT (Tissue-Tek, Sakura Finetek) medium and stored at −20 °C for over 2 h before sectioning. Sections (10 µm thick) were cut at −20 °C using a Leica CM1850 cryostat (Leica) and mounted on microscope slides (Fisher Scientific) at room temperature. Images were collected using a Zeiss AxioImager M1 fluorescence microscope equipped with a Zeiss Axiocam 712 colour CCD camera and plan-apochromat ×5 (NA = 0.5) objective, using ZEN 3.2 acquisition software (Carl Zeiss). All images were processed using Photoshop CC (Adobe).

### The parent-of-origin analysis of asexual embryo

The embryos from the double heterozygous mutants pollinated with *indica* rice 9311 were isolated for DNA extraction individually. A pair of primers flanking an SNP at *OsYUCCA11* (ref. ^32^) between Nipponbare (Nip) and 9311 was used to amplify DNA isolated from the aborted embryos. The PCR products were used for Sanger sequencing.

Autonomous seeds were harvested, sterilized in 20% bleach and washed 5 times with sterilized water. The asexual embryos were dissected out and used for callus induction using N6D medium^64^. The induced calli (~4 weeks induction) and internal control leaves of *Bellis prennis* (50 mg) were placed in a Petri dish on ice. The samples were gently chopped in 500 μl of modified Galbraith’s buffer^68^ for 30 s and gently mixed. After adding another 500 μl buffer, the samples were filtered through a two-step filter (42 µm first, then 20 µm) and collected in a flow cytometry sample cup. RNase (50 μl, 10 mg ml^−1^), 50 μl propidium iodide stock (1 mg ml^−1^) and 2 μl beta-mercapethanol were added to each sample for flow cytometry assay (Beckman Coulter). The DNA content and ploidy level were analysed^30,31^.

### RNA isolation and transcriptomic analysis

Asexual embryos and autonomous endosperm from ~20 ovules at 9 d post emasculation were harvested and collected in 1.5 ml RNase-free Eppendorf tubes for RNA isolation using TRIzol (Thermo Fisher) on ice. RNA quality was assessed using the Agilent Bioanalyzer with RNA integrity number values over 7. Freeze-dried triplicate RNA samples were sent to Novogene for transcriptome sequencing on an Illumina platform. The reads for the six transcriptomic data were deposited in NCBI under BioProject PRJNA786704. The public transcriptome data were downloaded from the NCBI FTP site (Supplementary Table 3). All data were aligned to the reference genome sequence of *O.* *sativa* ssp. *japonica* cv. Nipponbare^69^ (MSU 7.0) using HISAT (v.2.2.0)^70^. The alignment results were output as bam files. Samtools (v.0.1.19)^71^ was used to sort and index the bam files containing the aligned reads. The alignments were visualized using IGV genome browser^72^. The reference genome mapping ratio of the alignments was also counted by Samtools (Supplementary Table 3). Read counts were generated by featureCounts (v.2.0.1)^73^. The gene expression value was calculated using the transcript per million (TPM) method, which was based on reads counts and transcript length. After log_2_ normalization, heat mapping and clustering analysis were performed using TBtools^74^. PCA was performed using the online platform Majorbio Cloud (www.majorbio.com)^75^.

On the basis of the RNA-seq raw counts, differentially expressed genes (DEGs) analysis was performed using DESeq2 (ref. ^76^) in the R package for comparison between asexual embryo and sexual embryo, and between asexual endosperm and sexual endosperm. Genes with |log_2_(fold change)| > 2 and adjusted *P* value (*P*_adj_) < 0.01 were selected as DEGs (Supplementary Tables 4 and 7). DEGs were visualized using volcano plots in TBtools, which effectively displays the significance against the log_2_ fold change of the genes and highlights genes that are most differentially expressed (Extended Data Fig. 7a,b).

GO annotation of the whole genome was downloaded from Biomart (http://www.biomart.org). Biological Network Gene Ontology (BiNGO v.3.0.5)^77^, a Cytoscape^78^ plugin, was used to analyse GO enrichment and display the GO network diagram. Enrichment significance was determined using a hypergeometric test, with terms having a corrected *P* value below 0.05 being considered as enriched (Supplementary Tables 5 and 8).

### Reporting summary

Further information on research design is available in the Nature Portfolio Reporting Summary linked to this article.

### Supplementary information

Supplementary Information Supplementary Method for ChIP assay.

Reporting Summary

Supplementary Tables Supplementary Tables 1–18.

### Source data

**Source Data Fig. 5a**. Data for frequency of asexual embryo and autonomous seed formation at different stages. **Source Data Fig. 5g, Source Data Extended Data Fig. 6c, Source Data Extended Data Fig. 7c–k and Source Data Extended Data Fig. 8b**. Transcriptomic data for relevant gene expression. **Source Data Fig. 6a**. Data for heat map showing expression levels of *BBM1* and *WUS* family genes in asexual embryos and other tissues.

## Data Availability

Transcriptomic and ChIP-seq data generated in this study are deposited in the NCBI database under BioProject PRJNA786704, accession numbers: SRR17151221, SRR17151220, SRR17151219, SRR17151224, SRR17151223, SRR17151222, SRR17210911, SRR17210910, SRR25655515, SRR25655516, SRR25678596, SRR25678595. Other transcriptomic data used in the analysis were downloaded from NCBI including the BioProject PRJNA218883 (accession numbers: SRR976336, SRR976337, SRR976338, SRR976339, SRR976340, SRR976341, SRR976335, SRR976342, SRR976343), BioProject PRJNA295002 (accession numbers: SRR2295903, SRR2295904, SRR2295905, SRR2295906, SRR2295907, SRR2295908), BioProject PRJNA412710 (accession numbers: SRR6122716, SRR6122707, SRR6122710, SRR6122706, SRR6122708, SRR6122722, SRR6122709, SRR6122720, SRR6122704, SRR6122715), BioProject PRJDA51201 (accession number: DRR000623). Source data are provided with this paper.

## Code availability

Scripts specifically for the integrated data analysis of ChIP-seq datasets and SNP bias in hybrid rice from endosperm from the reciprocal crosses between Nipponbare (Nip) and 9311 are available on GitHub (https://github.com/biz007/NPLANTS-211112076-CSIRO).

## References

[1] Dresselhaus T, Jurgens G (2021). Comparative embryogenesis in angiosperms: activation and patterning of embryonic cell lineages. Annu. Rev. Plant Biol..

[2] Palovaara J, de Zeeuw T, Weijers D (2016). Tissue and organ initiation in the plant embryo: a first time for everything. Annu. Rev. Cell Dev. Biol..

[3] Anderson SN (2017). The zygotic transition is initiated in unicellular plant zygotes with asymmetric activation of parental genomes. Dev. Cell.

[4] Khanday I, Skinner D, Yang B, Mercier R, Sundaresan V (2019). A male-expressed rice embryogenic trigger redirected for asexual propagation through seeds. Nature.

[5] Kwong RW (2003). LEAFY COTYLEDON1-LIKE defines a class of regulators essential for embryo development. Plant Cell.

[6] Conner JA, Mookkan M, Huo H, Chae K, Ozias-Akins P (2015). A parthenogenesis gene of apomict origin elicits embryo formation from unfertilized eggs in a sexual plant. Proc. Natl Acad. Sci. USA.

[7] Chen B (2022). BABY BOOM regulates early embryo and endosperm development. Proc. Natl Acad. Sci. USA.

[8] Chaudhury, A. M. et al. Fertilization-independent seed development in *Arabidopsis thaliana*. *Proc. Natl Acad. Sci. USA* **94**, 4223–4228 (1997). 10.1073/pnas.94.8.4223 PMC20611 9108133

[9] Ohad N (1999). Mutations in FIE, a WD polycomb group gene, allow endosperm development without fertilization. Plant Cell.

[10] Kohler, C. et al. *Arabidopsis* MSI1 is a component of the MEA/FIE Polycomb group complex and required for seed development. *EMBO J.* **22**, 4804–4814 (2003). 10.1093/emboj/cdg444 PMC212713 12970192

[11] Luo, M. et al. Genes controlling fertilization-independent seed development in *Arabidopsis thaliana*. *Proc. Natl Acad. Sci. USA* **96**, 296–301 (1999). 10.1073/pnas.96.1.296 PMC15133 9874812

[12] Kiyosue, T. et al. Control of fertilization-independent endosperm development by the *MEDEA* polycomb gene in *Arabidopsis*. *Proc. Natl Acad. Sci. USA* **96**, 4186–4191 (1999). 10.1073/pnas.96.7.4186 PMC22442 10097185

[13] Luo, M., Bilodeau, P., Dennis, E. S., Peacock, W. J. & Chaudhury, A. Expression and parent-of-origin effects for *FIS2*, *MEA*, and *FIE* in the endosperm and embryo of developing *Arabidopsis* seeds. *Proc. Natl Acad. Sci. USA* **97**, 10637–10642 (2000). 10.1073/pnas.170292997 PMC27077 10962025

[14] Cheng, X. et al. Functional divergence of two duplicated *Fertilization Independent Endosperm* genes in rice with respect to seed development. *Plant J.* **104**, 124–137 (2020). 10.1111/tpj.14911 33463824

[15] Tonosaki, K. et al. Mutation of the imprinted gene *OsEMF2a* induces autonomous endosperm development and delayed cellularization in rice. *Plant Cell* **33**, 85–103 (2021). 10.1093/plcell/koaa006 PMC8136911 33751094

[16] Figueiredo DD, Batista RA, Roszak PJ, Kohler C (2015). Auxin production couples endosperm development to fertilization. Nat. Plants.

[17] Derkacheva, M. & Hennig, L. Variations on a theme: Polycomb group proteins in plants. *J. Exp. Bot.* **65**, 2769–2784 (2014). 10.1093/jxb/ert410 24336446

[18] Hsieh, T.-F. et al. Regulation of imprinted gene expression in *Arabidopsis* endosperm. *Proc. Natl Acad. Sci. USA* **108**, 1755–1762 (2011). 10.1073/pnas.1019273108 PMC3033266 21257907

[19] Wolff P (2011). High-resolution analysis of parent-of-origin allelic expression in the *Arabidopsis* endosperm. PLoS Genet..

[20] Weinhofer I, Hehenberger E, Roszak P, Hennig L, Kohler C (2010). H3K27me3 profiling of the endosperm implies exclusion of polycomb group protein targeting by DNA methylation. PLoS Genet..

[21] Moreno-Romero J, Jiang H, Santos-Gonzalez J, Kohler C (2016). Parental epigenetic asymmetry of PRC2-mediated histone modifications in the *Arabidopsis* endosperm. EMBO J..

[22] Zhang M (2014). Genome-wide high resolution parental-specific DNA and histone methylation maps uncover patterns of imprinting regulation in maize. Genome Res..

[23] Dong X (2017). Dynamic and antagonistic allele-specific epigenetic modifications controlling the expression of imprinted genes in maize endosperm. Mol. Plant.

[24] Figueiredo, D. D. & Köhler, C. Auxin: a molecular trigger of seed development. *Genes Dev.* **32**, 13 479–490 (2018). 10.1101/gad.312546.118 PMC5959232 29692356

[25] Luo M, Platten D, Chaudhury A, Peacock WJ, Dennis ES (2009). Expression, imprinting, and evolution of rice homologs of the polycomb group genes. Mol. Plant.

[26] Nallamilli, B. R. R. et al. Polycomb group gene *OsFIE2* regulates rice (*Oryza sativa*) seed development and grain filling via a mechanism distinct from *Arabidopsis*. *PLoS Genet.* **9**, e1003322 (2013). 10.1371/journal.pgen.1003322 PMC3591265 23505380

[27] Li, S. et al. *OsFIE2* plays an essential role in the regulation of rice vegetative and reproductive development. *New Phytol.* **201**, 66–79 (2014). 10.1111/nph.12472 24020752

[28] Maruyama D (2015). Rapid elimination of the persistent synergid through a cell fusion mechanism. Cell.

[29] You L (2021). Identification and analysis of genes involved in double fertilization in rice. Int. J. Mol. Sci..

[30] Dolezel J, Greilhuber J, Suda J (2007). Estimation of nuclear DNA content in plants using flow cytometry. Nat. Protoc..

[31] Cousin, A., Heel, K., Cowling, W. A. & Nelson, M. N. An efficient high-throughput flow cytometric method for estimating DNA ploidy level in plants. *Cytometry A* **75**, 1015–1019 (2009). 10.1002/cyto.a.20816 19845019

[32] Luo M (2011). A genome-wide survey of imprinted genes in rice seeds reveals imprinting primarily occurs in the endosperm. PLoS Genet..

[33] Anderson SN (2013). Transcriptomes of isolated *Oryza sativa* gametes characterized by deep sequencing: evidence for distinct sex-dependent chromatin and epigenetic states before fertilization. Plant J..

[34] Indoliya Y (2016). Decoding regulatory landscape of somatic embryogenesis reveals differential regulatory networks between *japonica* and *indica* rice subspecies. Sci. Rep..

[35] Sato, Y. et al. A rice homeobox gene, OSH1, is expressed before organ differentiation in a specific region during early embryogenesis. *Proc. Natl Acad. Sci*. *USA* **93**, 8117–8122 (1996). 10.1073/pnas.93.15.8117 PMC38885 8755613

[36] Sentoku, N. et al. Regional expression of the rice *KN1*-type homeobox gene family during embryo, shoot, and flower development. *Plant Cell* **11**, 1651–1663 (1999). 10.1105/tpc.11.9.1651 PMC144314 10488233

[37] Sato, Y., Sentoku, N., Nagato, Y. & Matsuoka, M. Isolation and characterization of a rice homebox gene, OSH15. *Plant Mol. Biol.* **38**, 983–998 (1998). 10.1023/a:1006065622251 9869405

[38] Ito M (2002). Position dependent expression of GL2-type homeobox gene, Roc1: significance for protoderm differentiation and radial pattern formation in early rice embryogenesis. Plant J..

[39] Huang, X., Peng, X. & Sun, M.-X. *OsGCD1* is essential for rice fertility and required for embryo dorsal-ventral pattern formation and endosperm development. *New Phytol.* **215**, 1039–1058 (2017). 10.1111/nph.14625 28585692

[40] Yi, J. et al. *OsMPK6* plays a critical role in cell differentiation during early embryogenesis in *Oryza sativa*. *J. Exp. Bot.* **67**, 2425–2437 (2016). 10.1093/jxb/erw052 PMC4809295 26912801

[41] Kamiya, N. et al. Rice *globular embryo 4* (*gle4*) mutant is defective in radial pattern formation during embryogenesis. *Plant Cell Physiol*. **44**, 875–883 (2003). 10.1093/pcp/pcg112 14519768

[42] Ito, Y., Eiguchi, M. & Kurata, N. Expression of novel homeobox genes in early embryogenesis in rice. *Biochim. Biophys. Acta* **1444**, 445–450 (1999). 10.1016/s0167-4781(99)00023-8 10095070

[43] Horst NA (2016). A single homeobox gene triggers phase transition, embryogenesis and asexual reproduction. Nat. Plants.

[44] Yao, L. et al. *OsMATL* mutation induces haploid seed formation in *indica* rice. *Nat. Plants* **4**, 530–533 (2018). 10.1038/s41477-018-0193-y 29988153

[45] Conner JA, Podio M, Ozias-Akins P (2017). Haploid embryo production in rice and maize induced by PsASGR-BBML transgenes. Plant Reprod..

[46] Cheng, X. et al. The maternally expressed polycomb group gene *OsEMF2a* is essential for endosperm cellularization and imprinting in rice. *Plant Commun.* **2**, 100092 (2021). 10.1016/j.xplc.2020.100092 PMC7816080 33511344

[47] Gehring M, Missirian V, Henikoff S (2011). Genomic analysis of parent-of-origin allelic expression in *Arabidopsis thaliana* seeds. PLoS ONE.

[48] Du, M., Luo, M., Zhang, R., Finnegan, E. J. & Koltunow, A. M. G. Imprinting in rice: the role of DNA and histone methylation in modulating parent-of-origin specific expression and determining transcript start sites. *Plant J.* **79**, 232–242 (2014). 10.1111/tpj.12553 24819479

[49] Waters AJ (2013). Comprehensive analysis of imprinted genes in maize reveals allelic variation for imprinting and limited conservation with other species. Proc. Natl Acad. Sci. USA.

[50] Hater F, Nakel T, Gross-Hardt R (2020). Reproductive multitasking: the female gametophyte. Annu. Rev. Plant Biol..

[51] Nowack MK (2006). A positive signal from the fertilization of the egg cell sets off endosperm proliferation in angiosperm embryogenesis. Nat. Genet..

[52] Guitton, A.-E. & Berger, F. Loss of function of MULTICOPY SUPPRESSOR OF IRA 1 produces nonviable parthenogenetic embryos in *Arabidopsis*. *Curr. Biol.* **15**, 750–754 (2005). 10.1016/j.cub.2005.02.066 15854908

[53] Mosquna A (2009). Regulation of stem cell maintenance by the polycomb protein FIE has been conserved during land plant evolution. Development.

[54] Okano, Y. et al. A polycomb repressive complex 2 gene regulates apogamy and gives evolutionary insights into early land plant evolution. *Proc. Natl Acad. Sci. USA* **106**, 16321–16326 (2009). 10.1073/pnas.0906997106 PMC2752547 19805300

[55] Boutilier K (2002). Ectopic expression of BABY BOOM triggers a conversion from vegetative to embryonic growth. Plant Cell.

[56] Lowe, K. et al. Morphogenic regulators *Baby boom* and *Wuschel* improve monocot transformation. *Plant Cell* **28**, 1998–2015 (2016). 10.1105/tpc.16.00124 PMC5059793 27600536

[57] Zhang, T.-Q. et al. A two-step model for de novo activation of *WUSCHEL* during plant shoot regeneration. *Plant Cell* **29**, 1073–1087 (2017). 10.1105/tpc.16.00863 PMC5466026 28389585

[58] Dunwell JM (2010). Haploids in flowering plants: origins and exploitation. Plant Biotechnol. J..

[59] Hand, M. L. & Koltunow, A. M. G. The genetic control of apomixis: asexual seed formation. *Genetics* **197**, 441–450 (2014). 10.1534/genetics.114.163105 PMC4063905 24939990

[60] Ozias-Akins P, van Dijk PJ (2007). Mendelian genetics of apomixis in plants. Annu. Rev. Genet.

[61] Khush, G. S. (ed.) *Apomixis: Exploiting Hybrid Vigor in Rice* (International Rice Research Institute, 1994).

[62] Wang K (2019). Fixation of hybrid vigor in rice: synthetic apomixis generated by genome editing. aBIOTECH.

[63] Ma X (2015). A robust CRISPR/Cas9 system for convenient, high-efficiency multiplex genome editing in monocot and dicot plants. Mol. Plant.

[64] Hiei Y, Komari T (2008). *Agrobacterium*-mediated transformation of rice using immature embryos or calli induced from mature seed. Nat. Protoc..

[65] Wu, X., Liu, J., Li, D. & Liu, C.-M. Rice caryopsis development II: dynamic changes in the endosperm. *J. Integr. Plant Biol.* **58**, 786–798 (2016). 10.1111/jipb.12488 27449987

[66] Zeng, Y.-X., Hu, C.-Y., Lu, Y.-G., Li, J.-Q. & Liu, X.-D. Abnormalities occurring during female gametophyte development result in the diversity of abnormal embryo sacs and leads to abnormal fertilization in *indica*/*japonica* hybrids in rice. *J. Integr. Plant Biol.* **51**, 3–12 (2009). 10.1111/j.1744-7909.2008.00733.x 19166488

[67] Morrison, W. R. & Laignelet, B. An improved colorimetric procedure for determining apparent and total amylose in cereal and other starches. *J. Cereal Sci.* **1**, 9–20 (1983).

[68] Galbraith DW (1983). Rapid flow cytometric analysis of the cell cycle in intact plant tissues. Science.

[69] Kawahara Y (2013). Improvement of the *Oryza sativa* Nipponbare reference genome using next generation sequence and optical map data. Rice.

[70] Kim D, Paggi JM, Park C, Bennett C, Salzberg SL (2019). Graph-based genome alignment and genotyping with HISAT2 and HISAT-genotype. Nat. Biotechnol..

[71] Danecek P (2021). Twelve years of SAMtools and BCFtools. Gigascience.

[72] Robinson JT, Thorvaldsdottir H, Wenger AM, Zehir A, Mesirov JP (2017). Variant review with the Integrative Genomics Viewer. Cancer Res..

[73] Liao Y, Smyth GK, Shi W (2014). featureCounts: an efficient general purpose program for assigning sequence reads to genomic features. Bioinformatics.

[74] Chen C (2020). TBtools: an integrative toolkit developed for interactive analyses of big biological data. Mol. Plant.

[75] Ren, Y. et al. Majorbio Cloud: a one-stop, comprehensive bioinformatic platform for multiomics analyses. *iMeta*10.1002/imt2.12 (2022). 10.1002/imt2.12 PMC10989754 38868573

[76] Love MI, Huber W, Anders S (2014). Moderated estimation of fold change and dispersion for RNA-seq data with DESeq2. Genome Biol..

[77] Maere S, Heymans K, Kuiper M (2005). BiNGO: a Cytoscape plugin to assess overrepresentation of gene ontology categories in biological networks. Bioinformatics.

[78] Shannon P (2003). Cytoscape: a software environment for integrated models of biomolecular interaction networks. Genome Res..

