# Supplementary material for: Mutation in Polycomb repressive complex 2 gene OsFIE2 promotes asexual embryo formation in rice
Source: Nat Plants. 2023 Oct 9;9(11):1848–61. doi: 10.1038/s41477-023-01536-4 (PMC10654051; doi:10.1038/s41477-023-01536-4)
Supplement: Supplementary file 1 — Supplementary Method for ChIP assay. [file 41477_2023_1536_MOESM1_ESM.pdf]

nature plants

Article https://doi.org/10.1038/s41477-023-01536-4

# Mutation in Polycomb repressive complex 2 gene *OsFIE2* promotes asexual embryo formation in rice

In the format provided by the authors and unedited

**Chromatin Immunoprecipitation assay of $H3K27me^3$ marks in endosperm**

To understand how $H3K27me^3$ (histone H3 lysine27 tri-methylation) catalysed by PRC2 associates with the PEGs in the rice endosperm, we used chromatin immunoprecipitation with anti-body against $H3K27me^3$ followed by deep sequencing (ChIP-seq) to identify $H3K27me^3$ marked regions in the maternal or paternal genomes of endosperm from the reciprocal crosses between Nipponbare (Nip) and 9311. The endosperm from two reciprocal crosses between the Japonica rice subspecies Nip and the Chinese Indica subspecies 9311 were isolated in a previous study[1]. The 2 grams of endosperm at 6 DAF were used to extract chromatins and Chromatin Immunoprecipitation (ChIP) assay using antibody (Millipore, 07-449) against $H3K27me^3$ were performed as described by Cui et al[2]. ChIP-seq libraries for Illumina single-end sequencing were prepared using the NEBNext DNA Library Prep Master Mix Set for Illumina (New England BioLabs, E6040S) according to the manufacturer's protocol. The ChIP-seq reads from the Nip and 9311 crosses were deposited in NCBI. The reads were mapped to the Nip and 9311 reference genomes (MSU7) as previously described (https://doi.org/10.1111/nph.15820), and the reads carrying SNPs were called by the SAMtools (v0.1.19) (https://doi.org/10.1093/bioinformatics/btp352) and BCFtools (v0.1.19) (https://doi.org/10.1093/bioinformatics/btr509). $H3K27me^3$ enriched regions (or peaks) were identified by MACS (v1.4.3)[3]. For verification of the paternal or maternal bias of $H3K27me^3$ enrichment, input DNA and ChIP DNA by antibody against Histone 3, and $H3K27me^3$ were isolated to perform qPCR using a pair of primer flanking the region of SNPs at *OsYUCCA11*. The qPCR products were sequenced using Sanger method and visualized to investigate the parental enrichment of $H3K27me^3$. PCR was also performed for other five PEGs followed by Sanger sequencing to visualize the enrichment between parental alleles. The trace data from sanger sequencing was visualized with Finch TV 1.4.0.

In total ~40 million and 16 million reads respectively from 9311xNip and Nipx9311 were mapped to the reference genome. We calculated the maternal or paternal reads based on the SNPs between the two genomes and identified 11565 maternally biased and 952 paternally biased SNPs (Supplementary Table 14). Using those SNPs, we identified parentally biased peaks targeted by $H3K27me^3$ (Extended Data Figure 8a). The number of maternally biased peaks (3602) is significantly more than that of paternally biased peaks (94), suggesting that $H3K27me^3$ preferentially targets the maternal genome in endosperm (Extended Data Figure 8a), consistent with the observations in Arabidopsis and maize. Among the 683 genes associated with the maternally biased SNPs (Supplementary Table 17), 36 are PEGs and only three are MEGs; among 87 genes associated with paternally biased peaks, two are imprinted genes (one PEG and one MEG)[30] (Extended Data Figure 8c,d; Supplementary Table 15-17), suggesting maternally biased $H3K27me^3$ silenced the maternal alleles of PEGs. Quantitative PCR performed for the PEG *OsYUCCA11* with maternally biased SNPs on the ChIP DNAs verified the histone modification (Extended Data Figure 8b-d). Furthermore, Sanger sequencing on PCR products amplified from several $H3K27me^3$ targeted PEGs on the ChIP DNAs confirmed that the maternal alleles are marked by $H3K27me^3$ (Extended Data Figure 9a,b). Many of these $H3K27me^3$-associated PEGs are also activated in autonomous endosperm (Figure 6c), suggesting that the expression of PEGs in the autonomous endosperm was likely caused by the loss of $H3K27me^3$ at the maternal alleles due to the *Osfie* mutations.

1 Luo, M. *et al.* A genome-wide survey of imprinted genes in rice seeds reveals imprinting primarily occurs in the endosperm. *PLoS Genet* **7**, e1002125 (2011). https://doi.org:10.1371/journal.pgen.1002125
2 Cui, X. *et al.* REF6 recognizes a specific DNA sequence to demethylate H3K27me3 and regulate organ boundary formation in Arabidopsis. *Nat Genet* **48**, 694-699 (2016). https://doi.org:10.1038/ng.3556
3 Zhang, Y. *et al.* Model-based analysis of ChIP-Seq (MACS). *Genome Biol* **9**, R137 (2008). https://doi.org:10.1186/gb-2008-9-9-r137
4 Hsieh, T. F. *et al.* Regulation of imprinted gene expression in Arabidopsis endosperm. *Proc Natl Acad Sci U S A* **108**, 1755-1762 (2011). https://doi.org:10.1073/pnas.1019273108
5 Weinhofer, I., Hehenberger, E., Roszak, P., Hennig, L. & Kohler, C. H3K27me3 profiling of the endosperm implies exclusion of polycomb group protein targeting by DNA methylation. *PLoS Genet* **6** (2010). https://doi.org:10.1371/journal.pgen.1001152
6 Moreno-Romero, J., Jiang, H., Santos-Gonzalez, J. & Kohler, C. Parental epigenetic asymmetry of PRC2-mediated histone modifications in the Arabidopsis endosperm. *EMBO J* **35**, 1298-1311 (2016). https://doi.org:10.15252/embj.201593534
7 Zhang, M. *et al.* Genome-wide high resolution parental-specific DNA and histone methylation maps uncover patterns of imprinting regulation in maize. *Genome Res* **24**, 167-176 (2014). https://doi.org:10.1101/gr.155879.113
8 Dong, X. *et al.* Dynamic and Antagonistic Allele-Specific Epigenetic Modifications Controlling the Expression of Imprinted Genes in Maize Endosperm. *Mol Plant* **10**, 442-455 (2017). https://doi.org:10.1016/j.molp.2016.10.007
