# Supplementary material for: Mutation in Polycomb repressive complex 2 gene OsFIE2 promotes asexual embryo formation in rice
Source: Nat Plants. 2023 Oct 9;9(11):1848–61. doi: 10.1038/s41477-023-01536-4 (PMC10654051; doi:10.1038/s41477-023-01536-4)
Supplement: Supplementary file 2 — Reporting Summary [file 41477_2023_1536_MOESM2_ESM.pdf]

nature portfolio

Corresponding author(s): Ming Luo and Xiaoba Wu

Last updated by author(s): 2023-8-30

Reporting Summary

Nature Portfolio wishes to improve the reproducibility of the work that we publish. This form provides structure for consistency and transparency in reporting. For further information on Nature Portfolio policies, see our Editorial Policies and the Editorial Policy Checklist.

Statistics

For all statistical analyses, confirm that the following items are present in the figure legend, table legend, main text, or Methods section.

n/a	Confirmed	
☐	☒	The exact sample size (*n*) for each experimental group/condition, given as a discrete number and unit of measurement
☐	☒	A statement on whether measurements were taken from distinct samples or whether the same sample was measured repeatedly
☐	☒	The statistical test(s) used AND whether they are one- or two-sided<br>*Only common tests should be described solely by name; describe more complex techniques in the Methods section.*
☒	☐	A description of all covariates tested
☒	☐	A description of any assumptions or corrections, such as tests of normality and adjustment for multiple comparisons
☐	☒	A full description of the statistical parameters including central tendency (e.g. means) or other basic estimates (e.g. regression coefficient) AND variation (e.g. standard deviation) or associated estimates of uncertainty (e.g. confidence intervals)
☐	☒	For null hypothesis testing, the test statistic (e.g. *F*, *t*, *r*) with confidence intervals, effect sizes, degrees of freedom and *P* value noted<br>*Give P values as exact values whenever suitable.*
☒	☐	For Bayesian analysis, information on the choice of priors and Markov chain Monte Carlo settings
☒	☐	For hierarchical and complex designs, identification of the appropriate level for tests and full reporting of outcomes
☒	☐	Estimates of effect sizes (e.g. Cohen's *d*, Pearson's *r*), indicating how they were calculated

Our web collection on statistics for biologists contains articles on many of the points above.

Software and code

Policy information about availability of computer code

Data collection	RNA-seq data were generated from Illumina HiSeq2500 platform with PE150 mode by service company Novogene. ChIP-seq libraries for Illumina single-end sequencing were prepared using the NEBNext DNA Library Prep Master Mix Set for Illumina (New England BioLabs, E6040S) according to the manufacturer's protocol. Confocal pictures were collected by Leica SP8 laser scanning confocal microscope (Leica Microsystems, Heidelberg, Germany). Other microscopy pictures were collect by Nikon COOLPIX dissection microscope or Zeiss AxioImager M1 fluorescence microscope. Cell Lab Quanta SC MPL flow cytometer (BECKMAN COULTER) was used for collecting DNA content data.
Data analysis	For transcriptome data analysis, all the data (we did or download from NCBI), the clean data were aligned to the reference genome sequence of Oryza.sativa ssp. Japonica cv. Nipponbare (MSU 7.0) using HISAT (version 2.2.0), For the following analysis steps, We used Samtools v0.1.19,FeatureCounts Version 2.0.158, DESeq R package,IGV (v2.7.0) geonme browser, TBtools v1.09832, BiNGO v3.0.5, Cytoscape v3.8.0.<br>ChIP data were aligned to two rice genome Nipponbare (MSU 7.0) and 9311. SNP called by SAMtools and BCFtools, the enriched peak were identified by MACS (V1.4.3).<br>Flow cytometric DNA histograms for ploidy levels were analyzed by CELL LAB QUANTA Collection Software (PC). SNP genotype were done by sanger sequencing and visualize by finch TV v1.4.0.

For manuscripts utilizing custom algorithms or software that are central to the research but not yet described in published literature, software must be made available to editors and reviewers. We strongly encourage code deposition in a community repository (e.g. GitHub). See the Nature Portfolio guidelines for submitting code & software for further information.

Data

Policy information about availability of data

All manuscripts must include a data availability statement. This statement should provide the following information, where applicable:

- Accession codes, unique identifiers, or web links for publicly available datasets
- A description of any restrictions on data availability
- For clinical datasets or third party data, please ensure that the statement adheres to our policy

Transcriptomic and ChIP-seq data generated in this study have been deposited in the NCBI database under accessionBioProject PRJNA786704, the accession numbers are: SRR17151221, SRR17151220, SRR17151219, SRR17151224, SRR17151223, SRR17151222, SRR17210911, and SRR17210910, SRR25655515, SRR25655516, SRR25678596, SRR25678595. These data are released and publicly available. Materials generated in this study are available upon request. Other Source transcriptomic data used in the analysis are provided with this paper were downloaded from NCBI public sites, these include the BioProject PRJNA218883 (the accession numbers are: SRR976336, SRR976337, SRR976338, SRR976339, SRR976340, SRR976341, SRR976335, SRR976342, SRR976343), BioProject PRJNA295002 (the accession numbers are: SRR2295903, SRR2295904, SRR2295905, SRR2295906, SRR2295907, SRR2295908), BioProject PRJNA412710 (the accession numbers are: SRR6122716, SRR6122707, SRR6122710, SRR6122706, SRR6122708, SRR6122722, SRR6122709, SRR6122720,SRR6122704, SRR6122715), BioProject PRJDA51201 (the accession numbers is DRR000623). Source data are provided.

Research involving human participants, their data, or biological material

Policy information about studies with human participants or human data. See also policy information about sex, gender (identity/presentation), and sexual orientation and race, ethnicity and racism.

Reporting on sex and gender	No Human participants involved
Reporting on race, ethnicity, or other socially relevant groupings	No Human participants involved
Population characteristics	No Human participants involved
Recruitment	No Human participants involved
Ethics oversight	No Human participants involved

Note that full information on the approval of the study protocol must also be provided in the manuscript.

Field-specific reporting

Please select the one below that is the best fit for your research. If you are not sure, read the appropriate sections before making your selection.

☒ Life sciences ☐ Behavioural & social sciences ☐ Ecological, evolutionary & environmental sciences

For a reference copy of the document with all sections, see nature.com/documents/nr-reporting-summary-flat.pdf

Life sciences study design

All studies must disclose on these points even when the disclosure is negative.

Sample size	Sample size was chosen based on what is commonly used in the filed. Samples size was considered sufficient to support the results. Transcription data have enough genome coverage with three biological repeats at least. For seed phenotypes, we used CRSPR/CAS9 to induce mutations in two genes independently and simultaneously. For each gene we randomly used three independent mutant lines to analyze the phenotype. For the double mutants, we used 4 random mutants to analyse the phenotype. for each mutant, the sample size is represented by ovule numbers used for scoring the novel phenotypes. We have stated the sample size in the figure legends or in the other places.
Data exclusions	We did not exclude any data for the novel phenotype, except for those initial observation at the first generation of transgenic plants when the CRSPR/Cas9 transgenes were present, which might affect the phenotyping. Therefore, for accurate phenotyping we need to segregate out the transgenes for stable phenotyping. We did start to collect complete data starting from generation T1 to T4.
Replication	We confirm all replication attempts were successful. For instance, to demonstrate the associated of the genotypes with phenotypes, we not only isolated 10 independent lines but also analyzed these lines in different generations. All the phenotypes were consistently observed between independently isolated mutant lines for each gene (3 repeats) or the double mutants (4 repeats). For transcriptomic data, three biological repeats were used. All attempts at replication were successful for transcriptomic data. For ChIP assay, two biological repeats were used. In the revised version, we moved the ChIP assay results into supplementary section as the results are consistent with previous finding and did not add much new in formation to the current results.
Randomization	All the plants and other samples were randomly assigned to the experimental groups. For example, plants with different genotypes were randomly selected for phenotyping. Sampling for ovules and seeds is randomly prepared for genotyping and phenotyping.

Blinding	Yes, we were blinded to conduct phenotyping on individual plants and then link the findings to the genetic analysis. Although samples were collected from plants with known genotype, the actual phenotype had to be scored under microscopy and there is no way to pre-exclude ovules for phenotyping. Therefore, we provided unbiased observation in this study.

Behavioural & social sciences study design

All studies must disclose on these points even when the disclosure is negative.

Study description	*Briefly describe the study type including whether data are quantitative, qualitative, or mixed-methods (e.g. qualitative cross-sectional, quantitative experimental, mixed-methods case study).*
Research sample	*State the research sample (e.g. Harvard university undergraduates, villagers in rural India) and provide relevant demographic information (e.g. age, sex) and indicate whether the sample is representative. Provide a rationale for the study sample chosen. For studies involving existing datasets, please describe the dataset and source.*
Sampling strategy	*Describe the sampling procedure (e.g. random, snowball, stratified, convenience). Describe the statistical methods that were used to predetermine sample size OR if no sample-size calculation was performed, describe how sample sizes were chosen and provide a rationale for why these sample sizes are sufficient. For qualitative data, please indicate whether data saturation was considered, and what criteria were used to decide that no further sampling was needed.*
Data collection	*Provide details about the data collection procedure, including the instruments or devices used to record the data (e.g. pen and paper, computer, eye tracker, video or audio equipment) whether anyone was present besides the participant(s) and the researcher, and whether the researcher was blind to experimental condition and/or the study hypothesis during data collection.*
Timing	*Indicate the start and stop dates of data collection. If there is a gap between collection periods, state the dates for each sample cohort.*
Data exclusions	*If no data were excluded from the analyses, state so OR if data were excluded, provide the exact number of exclusions and the rationale behind them, indicating whether exclusion criteria were pre-established.*
Non-participation	*State how many participants dropped out/declined participation and the reason(s) given OR provide response rate OR state that no participants dropped out/declined participation.*
Randomization	*If participants were not allocated into experimental groups, state so OR describe how participants were allocated to groups, and if allocation was not random, describe how covariates were controlled.*

Ecological, evolutionary & environmental sciences study design

All studies must disclose on these points even when the disclosure is negative.

Study description	*Briefly describe the study. For quantitative data include treatment factors and interactions, design structure (e.g. factorial, nested, hierarchical), nature and number of experimental units and replicates.*
Research sample	*Describe the research sample (e.g. a group of tagged Passer domesticus, all Stenocereus thurberi within Organ Pipe Cactus National Monument), and provide a rationale for the sample choice. When relevant, describe the organism taxa, source, sex, age range and any manipulations. State what population the sample is meant to represent when applicable. For studies involving existing datasets, describe the data and its source.*
Sampling strategy	*Note the sampling procedure. Describe the statistical methods that were used to predetermine sample size OR if no sample-size calculation was performed, describe how sample sizes were chosen and provide a rationale for why these sample sizes are sufficient.*
Data collection	*Describe the data collection procedure, including who recorded the data and how.*
Timing and spatial scale	*Indicate the start and stop dates of data collection, noting the frequency and periodicity of sampling and providing a rationale for these choices. If there is a gap between collection periods, state the dates for each sample cohort. Specify the spatial scale from which the data are taken*
Data exclusions	*If no data were excluded from the analyses, state so OR if data were excluded, describe the exclusions and the rationale behind them, indicating whether exclusion criteria were pre-established.*
Reproducibility	*Describe the measures taken to verify the reproducibility of experimental findings. For each experiment, note whether any attempts to repeat the experiment failed OR state that all attempts to repeat the experiment were successful.*
Randomization	*Describe how samples/organisms/participants were allocated into groups. If allocation was not random, describe how covariates were controlled. If this is not relevant to your study, explain why.*
Blinding	*Describe the extent of blinding used during data acquisition and analysis. If blinding was not possible, describe why OR explain why blinding was not relevant to your study.*

Did the study involve field work? ☐ Yes ☐ No

Field work, collection and transport

Field conditions	Plants were grown in enviroment-controlled glass houses
Location	*State the location of the sampling or experiment, providing relevant parameters (e.g. latitude and longitude, elevation, water depth).*
Access & import/export	*Describe the efforts you have made to access habitats and to collect and import/export your samples in a responsible manner and in compliance with local, national and international laws, noting any permits that were obtained (give the name of the issuing authority, the date of issue, and any identifying information).*
Disturbance	*Describe any disturbance caused by the study and how it was minimized.*

Reporting for specific materials, systems and methods

We require information from authors about some types of materials, experimental systems and methods used in many studies. Here, indicate whether each material, system or method listed is relevant to your study. If you are not sure if a list item applies to your research, read the appropriate section before selecting a response.

Materials & experimental systems

n/a	Involved in the study	
☐	☒	Antibodies
☒	☐	Eukaryotic cell lines
☒	☐	Palaeontology and archaeology
☒	☐	Animals and other organisms
☒	☐	Clinical data
☒	☐	Dual use research of concern
☐	☒	Plants

Methods

n/a	Involved in the study	
☐	☒	ChIP-seq
☐	☒	Flow cytometry
☒	☐	MRI-based neuroimaging

Antibodies

Antibodies used	Anti-trimethyl-Histone H3 (Lys27) (Know as H3K27me3), MilliporeSigma, Cat. 07-449, Rabbit Polyclonal Antibody, 1:200
Validation	Anti-trimethyl-Histone H3 (Lys27) was validated by the manufacturers and in publications lists of manufacturer's website (https://www.sigmaaldrich.com/AU/en/product/mm/07449)

Eukaryotic cell lines

Policy information about cell lines and Sex and Gender in Research

Cell line source(s)	*State the source of each cell line used and the sex of all primary cell lines and cells derived from human participants or vertebrate models.*
Authentication	*Describe the authentication procedures for each cell line used OR declare that none of the cell lines used were authenticated.*
Mycoplasma contamination	*Confirm that all cell lines tested negative for mycoplasma contamination OR describe the results of the testing for mycoplasma contamination OR declare that the cell lines were not tested for mycoplasma contamination.*
Commonly misidentified lines (See ICLAC register)	*Name any commonly misidentified cell lines used in the study and provide a rationale for their use.*

Palaeontology and Archaeology

Specimen provenance	*Provide provenance information for specimens and describe permits that were obtained for the work (including the name of the issuing authority, the date of issue, and any identifying information). Permits should encompass collection and, where applicable, export.*
Specimen deposition	*Indicate where the specimens have been deposited to permit free access by other researchers.*
Dating methods	*If new dates are provided, describe how they were obtained (e.g. collection, storage, sample pretreatment and measurement), where*

Dating methods | *they were obtained (i.e. lab name), the calibration program and the protocol for quality assurance OR state that no new dates are provided.*

☐ Tick this box to confirm that the raw and calibrated dates are available in the paper or in Supplementary Information.

Ethics oversight | *Identify the organization(s) that approved or provided guidance on the study protocol, OR state that no ethical approval or guidance was required and explain why not.*

Note that full information on the approval of the study protocol must also be provided in the manuscript.

Animals and other research organisms

Policy information about studies involving animals; ARRIVE guidelines recommended for reporting animal research, and Sex and Gender in Research

Laboratory animals	*For laboratory animals, report species, strain and age OR state that the study did not involve laboratory animals.*
Wild animals	*Provide details on animals observed in or captured in the field; report species and age where possible. Describe how animals were caught and transported and what happened to captive animals after the study (if killed, explain why and describe method; if released, say where and when) OR state that the study did not involve wild animals.*
Reporting on sex	*Indicate if findings apply to only one sex; describe whether sex was considered in study design, methods used for assigning sex. Provide data disaggregated for sex where this information has been collected in the source data as appropriate; provide overall numbers in this Reporting Summary. Please state if this information has not been collected. Report sex-based analyses where performed, justify reasons for lack of sex-based analysis.*
Field-collected samples	*For laboratory work with field-collected samples, describe all relevant parameters such as housing, maintenance, temperature, photoperiod and end-of-experiment protocol OR state that the study did not involve samples collected from the field.*
Ethics oversight	*Identify the organization(s) that approved or provided guidance on the study protocol, OR state that no ethical approval or guidance was required and explain why not.*

Note that full information on the approval of the study protocol must also be provided in the manuscript.

Clinical data

Policy information about clinical studies
All manuscripts should comply with the ICMJE guidelines for publication of clinical research and a completed CONSORT checklist must be included with all submissions.

Clinical trial registration	*Provide the trial registration number from ClinicalTrials.gov or an equivalent agency.*
Study protocol	*Note where the full trial protocol can be accessed OR if not available, explain why.*
Data collection	*Describe the settings and locales of data collection, noting the time periods of recruitment and data collection.*
Outcomes	*Describe how you pre-defined primary and secondary outcome measures and how you assessed these measures.*

Dual use research of concern

Policy information about dual use research of concern

Hazards

Could the accidental, deliberate or reckless misuse of agents or technologies generated in the work, or the application of information presented in the manuscript, pose a threat to:

No	Yes	
☒	☐	Public health
☒	☐	National security
☒	☐	Crops and/or livestock
☒	☐	Ecosystems
☒	☐	Any other significant area

Experiments of concern

Does the work involve any of these experiments of concern:

No	Yes	
☒	☐	Demonstrate how to render a vaccine ineffective
☒	☐	Confer resistance to therapeutically useful antibiotics or antiviral agents
☒	☐	Enhance the virulence of a pathogen or render a nonpathogen virulent
☒	☐	Increase transmissibility of a pathogen
☒	☐	Alter the host range of a pathogen
☒	☐	Enable evasion of diagnostic/detection modalities
☒	☐	Enable the weaponization of a biological agent or toxin
☒	☐	Any other potentially harmful combination of experiments and agents

Plants

Seed stocks

A common rice variety Nipponbare were used for transformation and gene editing. All the seeds were stored in PC2 lab located in CSIRO BLACK MOUNTAIN LABORATORY. The seeds are managed based on guidelines of The Office of Gene and Technology Regulation, Australia. The plants were growing in a PC2 glasshouses. The harvested seeds were double bagged during the transportation from glasshouse to PC2 lab.

Novel plant genotypes

Members of the conserved Polycomb Repressive Complex 2 (PRC2) OsFIE1 and OsFIE2 play important roles in plant development. We generated 3 Osfie1 and 3 Osfie2 single gene mutants , and 4 Osfie1/Osfie2 double mutant lines in rice strain Nipponbare with CRISPR/Cas9. The ovules of Osfie1 and Osfie2 double mutants exhibit asexual embryo and autonomous endosperm formation at a high frequency, while ovules of single Osfie2 mutants only display asexual pre-embryo-like structures at a lower frequency without fertilization. The asexual embryo formation induced by the Osfie2 mutation is a novel phenotypes which had not been described before in plants. In details, rice genome has two FIE homologs (LOC_Os08g04270 and LOC_Os08g04290) which are closely linked . The CRISPR/Cas9 editing method was used for mutant generation as previously described by Ma et al 2015. We cloned three sgRNA sequences in the transformation binary vector. The target seed sequences (~20bp) of the three sgRNAs were selected from the OsFIE1 (LOC_Os08g04290) and OsFIE2 (LOC_Os08g04270) coding regions, with two specifics for OsFIE1 (g3: GTCACCGACACGAAGTACT) and for OsFIE2 (g1: TCGTTCTACACACTGAGTT) respectively, and the third one targeting both genes (g2: CTCATCATTTCTGCAAGCA) (Figure 1 A). This will maximize the chance to generate mutations respectively and simultaneously at both closely linked loci. The rice small nuclear promoters OsU3, OsU6a and OsU6b were used to drive g1, g2 and g3 sgRNA respectively and the DNA for the three sgRNA expression were synthesized by IDT (Singapore) with Type II restriction enzyme Bsa I sites attached. These synthesized sequences were inserted into the binary vector pYLCRISPR/Cas9-MH (hosting SpCas9) using Goldengate strategy with Type II restriction enzyme Bsa I for digestion and T4 DNA ligase for ligation. The calli were induced from mature rice seeds (Oryza sativa ssp. japonica cv. Nipponbare) and transformation was performed by using Agrobacterium tumefaciens strain GV3101. Positive transformed calli were screened by hygromycin, then used to regenerate transgenic plants. In total ¬150 plants obtained. With random sequencing or primary phenotyping, 10 independent plants were obtained, with 4 showing aborted seeds formation (turned out to double mutants of the target Osfie1 and Osfie2 genes), 6 showing normal seed formation (3 were homozygote Osfie2 and 3 heterozygote of Osfie2.
The PCR products amplified from expected regions around mutations were directly used for Sanger sequencing and mutations were deduced from the sequencing traces. The transgene-free plants were isolated from above 10 primary lines to score the genotype and analyze phenotypes in subsequent generation such as T1,T2 or T4.

Authentication

To avoid the offtargeting effects, we have constructed all the guides targeting two genes on the same vector for transformation. In this way, all the independent lines (10 lines obtained in this study, 3 lines for gene1, 3 lines for gene2, 4 lines for double mutants) should borne the chance to have the phenotype, if off-targeting occurred. However, only lines with the gene2 mutations (7 lines) showed the expected phenotypes, indicating off-targeting is not responsible for the phenotype. Further analysis of the segregants from the 7 lines of the gene2 mutants showed that 35 plants of the progeny lacking of the gene2 mutations displayed no expected phenotypes, while the plants with the gene2 mutations showed the phenotype, supporting the offtargeting effect is negligible.

ChIP-seq

Data deposition

☒ Confirm that both raw and final processed data have been deposited in a public database such as GEO.

☒ Confirm that you have deposited or provided access to graph files (e.g. BED files) for the called peaks.

Data access links
May remain private before publication.

The data has been uploaded to NCBI and released

Files in database submission

NCBI BioProject: PRJNA786704

Genome browser session
(e.g. UCSC)

No applicable, no public genome browser like UCSC currently available to support rice genome and accept uploading BED files.

Methodology

Replicates	Two independent replicates
Sequencing depth	Over 30 million of raw reads with 100bp length, and about 62.2% (20.5 million) and 53.23% (16 million) were mapped to genome.
Antibodies	Anti-trimethyl-Histone H3 (Lys27) (Millipore Sigma, 07-449)
Peak calling parameters	ChIP-seq of H3K27me3 enriched regions (or peaks) were identified by MACS (v1.4.3) with default parameters.
Data quality	A total of 40 million and 16 million reads with two repeats respectively from 9311xNip and Nipx9311 were mapped to the reference genome. Those peaks of H3K27me3 identified with significant enrichment bias of parental or maternal alleles were based on the 2 fold changes of read counts after normalization of the paternal heterozygosity ratio of 2:1.
Software	Illumina (New England BioLabs, E6040S) according to the manufacturer's protocol to collect the data. SAMtools (v0.1.19) (https://doi.org/10.1093/bioinformatics/btp352) and BCFtools (v0.1.19) (https://doi.org/10.1093/bioinformatics/btr509) were used to do SNP call. H3K27me3 enriched regions (or peaks) were identified by MACS (v1.4.3)

Flow Cytometry

Plots

Confirm that:

☒ The axis labels state the marker and fluorochrome used (e.g. CD4-FITC).

☒ The axis scales are clearly visible. Include numbers along axes only for bottom left plot of group (a 'group' is an analysis of identical markers).

☒ All plots are contour plots with outliers or pseudocolor plots.

☒ A numerical value for number of cells or percentage (with statistics) is provided.

Methodology

Sample preparation	The calli and leave samples were chopped with a razor blade in 500ul of modified Galbraith's buffer for 30 seconds and gently mixed. After adding another 500ul buffer, the samples were filtered through a two-step filter (42 microns first and then 20 microns) and collected in a flow cytometry sample cup. 50ul RNase (10mg/ml), 50ul PI stock (1mg/ml) and 2ul beta-mercapethanol were added to each sample for flow cytometry assay.
Instrument	Beckman Coulter Cell Lab Quanta SC-MPL
Software	Cell Lab Quanta Analysis software
Cell population abundance	Calli and leaves were used for chopping. About 1000-7000 cells were counted to analysis
Gating strategy	The appropriate gating borders enclosing the florescence intensity peaks in the flow cytometric DNA histograms of tested samples exampled in Figure 4 B,C,D with internal controls were shown in the Extended Data Figure 5 (newly added in this revised version). Other biological samples were processed in the same way. All the data are now added as Supplementary Table 2. These examples of gating ensured to minimize the background noise.

☒ Tick this box to confirm that a figure exemplifying the gating strategy is provided in the Supplementary Information.

Magnetic resonance imaging

Experimental design

Design type	*Indicate task or resting state; event-related or block design.*
Design specifications	*Specify the number of blocks, trials or experimental units per session and/or subject, and specify the length of each trial or block (if trials are blocked) and interval between trials.*
Behavioral performance measures	*State number and/or type of variables recorded (e.g. correct button press, response time) and what statistics were used to establish that the subjects were performing the task as expected (e.g. mean, range, and/or standard deviation across subjects).*

Acquisition

Imaging type(s) — *Specify: functional, structural, diffusion, perfusion.*

Field strength — *Specify in Tesla*

Sequence & imaging parameters — *Specify the pulse sequence type (gradient echo, spin echo, etc.), imaging type (EPI, spiral, etc.), field of view, matrix size, slice thickness, orientation and TE/TR/flip angle.*

Area of acquisition — *State whether a whole brain scan was used OR define the area of acquisition, describing how the region was determined.*

Diffusion MRI ☐ Used ☐ Not used

Preprocessing

Preprocessing software — *Provide detail on software version and revision number and on specific parameters (model/functions, brain extraction, segmentation, smoothing kernel size, etc.).*

Normalization — *If data were normalized/standardized, describe the approach(es): specify linear or non-linear and define image types used for transformation OR indicate that data were not normalized and explain rationale for lack of normalization.*

Normalization template — *Describe the template used for normalization/transformation, specifying subject space or group standardized space (e.g. original Talairach, MNI305, ICBM152) OR indicate that the data were not normalized.*

Noise and artifact removal — *Describe your procedure(s) for artifact and structured noise removal, specifying motion parameters, tissue signals and physiological signals (heart rate, respiration).*

Volume censoring — *Define your software and/or method and criteria for volume censoring, and state the extent of such censoring.*

Statistical modeling & inference

Model type and settings — *Specify type (mass univariate, multivariate, RSA, predictive, etc.) and describe essential details of the model at the first and second levels (e.g. fixed, random or mixed effects; drift or auto-correlation).*

Effect(s) tested — *Define precise effect in terms of the task or stimulus conditions instead of psychological concepts and indicate whether ANOVA or factorial designs were used.*

Specify type of analysis: ☐ Whole brain ☐ ROI-based ☐ Both

Statistic type for inference — *Specify voxel-wise or cluster-wise and report all relevant parameters for cluster-wise methods.*

(See Eklund et al. 2016)

Correction — *Describe the type of correction and how it is obtained for multiple comparisons (e.g. FWE, FDR, permutation or Monte Carlo).*

Models & analysis

n/a	Involved in the study
☐	☐ Functional and/or effective connectivity
☐	☐ Graph analysis
☐	☐ Multivariate modeling or predictive analysis

Functional and/or effective connectivity — *Report the measures of dependence used and the model details (e.g. Pearson correlation, partial correlation, mutual information).*

Graph analysis — *Report the dependent variable and connectivity measure, specifying weighted graph or binarized graph, subject- or group-level, and the global and/or node summaries used (e.g. clustering coefficient, efficiency, etc.).*

Multivariate modeling and predictive analysis — *Specify independent variables, features extraction and dimension reduction, model, training and evaluation metrics.*
